# Scientific and Clinical Studies on Variation in Individual Response to Ionising Radiation: A Scoping Review

**DOI:** 10.7759/cureus.110674

**Published:** 2026-06-11

**Authors:** Abdul Nadim Asil, Lily Crouzier, Marc Benderitter, Christophe Badie

**Affiliations:** 1 Psychiatry, NHS England, London, GBR; 2 Medicine, Central and North West London NHS Foundation Trust, London, GBR; 3 Division of Health, Institut de Radioprotection et de Sûreté Nucléaire (IRSN), Paris, FRA; 4 Cancer Genetics and Cytogenetics, UK Health Security Agency (UKHSA), London, GBR

**Keywords:** ionising radiation, radiosensitivity, radiosusceptibility, radiotherapy (rt), response to radiation

## Abstract

Ionising radiation, defined as radiation with sufficient energy to remove electrons from atoms and molecules, can produce significant biological effects in living tissue. Individual response to ionising radiation varies considerably and underpins both deterministic tissue reactions and stochastic effects such as cancer. The concepts of radiosensitivity and radiosusceptibility are central to this variability; however, their definitions and applications remain inconsistent across the literature.

This scoping review aims to systematically map and analyse how radiosensitivity and radiosusceptibility are defined, applied, and distinguished in scientific and clinical studies of ionising radiation.

The review was conducted in accordance with the Arksey and O’Malley framework and reported using PRISMA-ScR guidelines. A comprehensive search of PubMed and Scopus (December 2025) was performed using a broad Boolean strategy to capture variation in terminology. Following staged filtering and duplicate removal, studies were screened at title/abstract and full-text levels by two independent reviewers using predefined inclusion and exclusion criteria. Data were extracted using a standardised framework and synthesised descriptively.

The search yielded over 14,000 records, which were systematically reduced through structured filtering to a manageable dataset for screening. The final included studies demonstrated substantial heterogeneity in study design, endpoints, and terminology. Radiosensitivity was predominantly characterised using cellular and molecular measures, including DNA damage, repair capacity, and cytogenetic assays, whereas radiosusceptibility was particularly associated with normal tissue toxicity and radiation-induced cancer risk. The term “radiosusceptibility” was comparatively underrepresented. Across studies, considerable inter-individual variability in radiation response was consistently observed.

This review highlights a lack of conceptual clarity and consistency in the use of radiosensitivity and radiosusceptibility. Greater definitional precision and methodological alignment are needed to improve comparability across studies and support the integration of biological and clinical evidence, with implications for personalised radiotherapy and radiation risk assessment.

## Introduction and background

Introduction

Types of Radiation

Ionising radiation refers to radiation possessing sufficient energy to remove electrons from atoms and molecules, thereby producing ionisation within biological tissues [1]. It exists in several forms with differing physical properties, penetration abilities, and biological effects. Particulate radiation includes alpha (α) particles, beta (β) particles, neutrons, and protons, whereas electromagnetic radiation includes X-rays and gamma (γ) rays. Alpha particles possess high linear energy transfer (LET) but limited penetration, making them particularly damaging when internalised. In contrast, beta particles demonstrate intermediate penetration and ionisation capacity, while gamma rays and X-rays are highly penetrating forms of low-LET radiation commonly encountered in medical imaging and radiotherapy [2]. These differing radiation qualities influence patterns of energy deposition, DNA damage, and subsequent biological response, contributing to variability in individual radiation effects.

Individual Response to Ionising Radiation

Advancements in radiobiology, alongside sustained research efforts from organisations such as the International Commission on Radiological Protection (ICRP), have significantly refined our understanding of the individual response to ionising radiation. This evolving body of work has elucidated complex biological mechanisms underlying radiation effects, including impacts on human health, distinctions between stochastic and non-stochastic effects, and the classification and characterisation of tissue reactions [2].

Early investigations into ionising radiation in the late 19th and early 20th centuries laid the foundation for the current understanding. Much of this initial knowledge was derived from anecdotal observations of harmful effects in early radiologists, patients undergoing X-ray treatments, and occupationally exposed groups such as the “Radium Girls” [3]. These early findings highlighted both the therapeutic potential and the significant risks associated with radiation exposure.

Stochastic effects of ionising radiation are those that occur by chance and whose probability is proportional to dose, with no threshold [4]. Additionally, the severity of these effects does not increase with dose. The primary stochastic effects are cancer and genetic mutations. The ICRP has extensively reviewed these effects and published guidelines on them. In ICRP Publication 103 [2], the ICRP elaborates on the linear no-threshold (LNT) model, which suggests that even the smallest dose has the potential to increase the risk of cancer and hereditary effects. This model is foundational for radiation protection policies worldwide.

In contrast, non-stochastic effects - more appropriately termed tissue reactions - are characterised by a dose threshold, below which effects are not expected to occur. Above this threshold, both the probability and severity of the effect increase with dose. Examples include skin erythema, radiation burns, and cataract formation. The ICRP has further elaborated on these effects in ICRP Publication 118 [5], providing updated threshold dose estimates and reinforcing the importance of dose limitation to prevent such outcomes.

Tissue reactions may be broadly categorised into early- and late-responding effects based on the time course of manifestation and underlying tissue characteristics [2]. Early tissue reactions typically occur within days to weeks following exposure and are associated with tissues exhibiting rapid cellular turnover. Common examples include skin erythema, nausea, vomiting, and gastrointestinal disturbances. The thresholds and severity of these reactions depend on factors such as radiation type, dose rate, and exposure duration [2].

Late tissue reactions, in contrast, may manifest months to years after exposure and are generally associated with tissues that have slower cellular turnover. These effects are often more severe and long-lasting and may include fibrosis, cataract formation, and cardiovascular complications such as ischaemic heart disease [2,5]. The manifestation and severity of late effects depend on multiple factors, including the irradiated volume, total dose, and individual biological variability.

Definition/Evolution of Terms

Radiosensitivity is a central concept in radiobiology and may be defined as the degree to which a biological entity is affected by exposure to ionising radiation. It primarily relates to immediate biological and tissue responses to radiation exposure, including DNA damage, cell death, and normal tissue reactions, and is therefore more closely associated with deterministic radiation effects [5]. It applies across multiple biological scales, from cells to tissues and whole organisms, and has important implications for radiation risk assessment, radiotherapy planning, and radiological protection. Radiosensitivity varies considerably depending on factors such as cell cycle phase, DNA repair capacity, oxygenation status, and intrinsic cellular characteristics [5].

A key early development in understanding radiosensitivity was the formulation of the Bergonie and Tribondeau law in 1906 [6]. This principle states that cells are more radiosensitive if they are actively dividing, less differentiated, and exhibit a high proliferative capacity. This provided a foundational explanation for why tissues with rapid turnover, such as bone marrow and the intestinal epithelium, are more affected by radiation than more differentiated tissues such as muscle and nerve [7]. Although this law remains broadly applicable, it has limitations and does not fully account for the behaviour of all cell types.

Further advances in the early 20th century were driven by pioneering experimental work. Muller demonstrated the mutagenic effects of X-rays in Drosophila in the 1920s, providing early evidence that genetic material is particularly vulnerable to radiation damage [8]. As radiobiology developed as a discipline, the concept of radiosensitivity expanded from empirical observations to more formalised frameworks. The ICRP has played a significant role in this progression. Since its 1977 recommendations and in subsequent publications, the ICRP has incorporated tissue-specific radiosensitivity into models used to derive dose limits and tissue weighting factors. These frameworks recognise that radiosensitivity varies not only between tissue types but also among individuals, thereby influencing radiation protection standards and practices [4].

Recent advances in molecular and cellular biology have further refined the understanding of radiosensitivity. Research into DNA damage response pathways, including double-strand break repair, apoptosis, and cell cycle regulation, has provided mechanistic insight into radiation-induced cellular damage. Studies of genes such as TP53 and BRCA1/2 have demonstrated how genetic variation can influence cellular responses to radiation [1]. These developments have contributed to the emergence of radiogenomics, which seeks to link genetic variation with radiation response, particularly in the context of personalised radiotherapy.

Overall, the concept of radiosensitivity has evolved from early empirical observations to a complex, multi-scale construct encompassing cellular-, tissue-, and individual-level responses.

While radiosensitivity is well established within radiobiology literature, the concept of radiosusceptibility is less consistently defined. Major sources such as Hall and Giaccia, UNSCEAR reports, and the BEIR VII report describe variability in radiation-induced cancer risk and genetic predisposition, which collectively underpin this concept, although the term itself is not uniformly adopted [1,9,10]. Radiosusceptibility may be understood as the predisposition of an individual to develop long-term adverse health effects, particularly stochastic outcomes such as cancer, following exposure to ionising radiation. It reflects the probability of long-term biological consequences rather than the immediate extent of cellular damage, operating at the level of whole organisms and populations and influenced by factors including genetic background, age, sex, lifestyle, and environmental exposures. As such, it encompasses the broader spectrum of radiation-induced health risks and outcomes [9].

Early indications of radiosusceptibility emerged from observational studies in the early 20th century, as researchers noted variability in outcomes following similar radiation exposures. Investigations across different organisms and tissue types suggested that certain individuals or species were more prone to radiation-induced genetic damage and subsequent disease. These findings contributed to the recognition that susceptibility to radiation-induced conditions, particularly cancer, is not uniform but varies across populations. Over time, modifying factors such as genetic background, age at exposure, sex, and environmental influences were identified as important determinants of this variability.

As the field progressed, the concept of radiosusceptibility became more formally embedded within radiation protection frameworks. The ICRP has played a central role in this development. In Publication 103 [2] and related reports, the Commission emphasises variability in individual risk, particularly through the concept of “cancer susceptibility”, which underpins population-based risk models. Although the term radiosusceptibility is not always explicitly used, it is conceptually derived from and closely aligned with this notion of differential cancer risk within populations.

In addition to ICRP publications, advancements in genetics and epidemiology have further refined the understanding of radiosusceptibility. Studies identifying specific genetic markers (e.g., mutations in BRCA1/2 and TP53) associated with increased cancer risk have highlighted the role of genetic predisposition in radiosusceptibility. Large-scale epidemiological studies, such as those involving atomic bomb survivors and radiation workers, have provided robust data on the long-term health effects of radiation exposure, contributing to a more nuanced understanding of radiosusceptibility [10].

**Figure 1:**
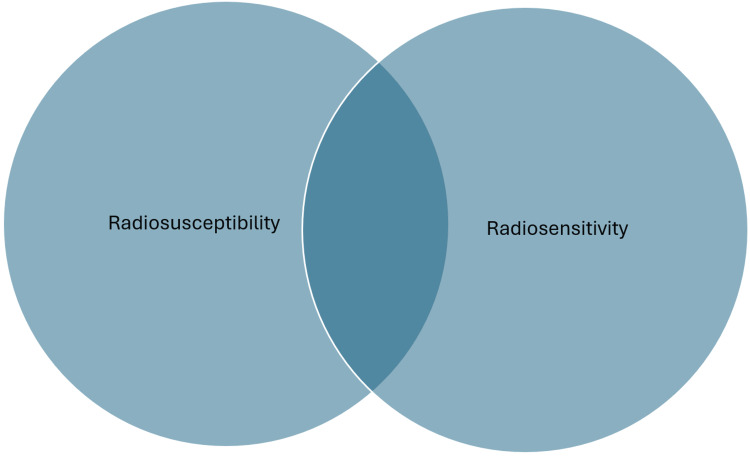
Overlap in concepts It is important to remember that these two concepts are distinctly defined, though they are predicted to have considerable overlap. Image Credit: Lily Crouzier

In summary, radiosusceptibility has evolved from early observational insights into a population-based and genetically informed concept that underpins radiation risk assessment. While it is closely related to radiosensitivity, radiosusceptibility is distinct in that it describes the likelihood of stochastic outcomes, such as cancer, rather than the severity of immediate biological damage. As illustrated in Figure 1, these concepts can be understood as overlapping but not synonymous domains: radiosensitivity primarily relates to tissue and cellular responses, whereas radiosusceptibility reflects longer-term health risks at the individual or population level. The area of overlap highlights shared biological mechanisms - such as DNA damage and repair pathways - that contribute to both immediate and long-term radiation effects.

Review Objectives

This scoping review aims to systematically map and analyse the use of the terms radiosensitivity and radiosusceptibility, focussing on the individual response within scientific and clinical studies of ionising radiation. While previous reviews have focused on specific radiation-induced outcomes, limited attention has been given to how these concepts are defined, applied, and interrelated. This conceptual ambiguity underscores the need for clearer differentiation and integration of these terms, forming a key focus of this scoping review. By employing clearly defined search terms and eligibility criteria, this review seeks to enhance conceptual clarity and identify gaps in the literature.

**Table 1:** Data extraction framework

Category	Data Extracted
Study Characteristics	Author(s), year of publication, journal, country of study
Study Design	Study design and methodology (e.g., clinical, in vivo, in vitro, epidemiological)
Population/Sample	Participant or sample characteristics (e.g., age, sex, health status, cell line, animal model)
Conceptual Definitions	Definitions and criteria used for radiosensitivity and/or radiosusceptibility
Key Findings	Main findings related to individual response to ionising radiation
Biomarkers	Identification and/or role of biomarkers associated with radiation response
Other Outcomes or Recommendations	Recommendations, frameworks, or implications for individual radiation risk assessment

## Review

Methodology

Frameworks

The methodology for this scoping review was conducted in accordance with the framework proposed by Arksey and O’Malley [11] and, where applicable, guided by the PRISMA extension for scoping reviews (PRISMA-ScR), as these approaches provide a structured yet flexible framework for systematically mapping broad and heterogeneous bodies of literature while ensuring transparency and reproducibility in reporting.

All identified records were screened in two stages: title and abstract screening, followed by full-text review. Two reviewers independently assessed studies against the predefined inclusion and exclusion criteria. Any disagreements were resolved through discussion and consensus, with a third reviewer consulted where necessary.

Database Search

A comprehensive literature search was conducted in December 2025 to identify relevant studies addressing variation in individual response to ionising radiation, with a specific focus on the concepts of radiosensitivity and radiosusceptibility.

The following electronic databases were searched: PubMed, Scopus. Initial keyword searches included terms such as “radiosensitivity,” “radiosusceptibility,” “individual response to ionising radiation,” “genetic factors in radiation response,” “DNA repair and radiation,” “radiotherapy and radiosensitivity,” and “radiation-induced cancer.”

Preliminary screening of these results highlighted substantial variability in the literature retrieved, which appeared sensitive to the specific phrasing of search terms. It was recognised that reliance on narrowly defined or highly specific keywords could introduce bias, as alternative phrasing (e.g., “genetic stability” versus “genetic instability” in radiation response, or variations of “DNA repair” such as “DNA damage” or “misrepair”) may yield different subsets of relevant studies. This demonstrated that the initial keyword list was not exhaustive and that overly prescriptive search terms risked limiting the scope of the review.

To address this, a more balanced and less restrictive search strategy was developed using Boolean operators. The final search strategy, upon which this review is based, is as follows: “Response to radiation” AND (“radiosensitivity” OR “radiation sensitivity” OR “radiosusceptibility” OR “radiation susceptibility”).

This approach was selected to maximise sensitivity while maintaining relevance to the core concepts of the review. A more restrictive alternative strategy, incorporating additional Boolean constraints, was considered: “Response to radiation” AND (“radiosensitivity” OR “radiation sensitivity” AND “radiosusceptibility” OR “radiation susceptibility”).

However, this approach substantially reduced the number of retrieved studies and was deemed overly restrictive, with the potential to exclude relevant literature. Where applicable, database search functions were utilised to automatically account for variations in terminology, including synonyms, truncated terms, and differences in spelling (e.g., British and American English).

During the initial screening phase, it was observed that the term “radiosusceptibility” yielded a limited number of results and, in some cases (e.g., PubMed), no direct matches. This finding supports the view that radiosusceptibility remains an emerging and relatively underrepresented concept within the literature. Existing reviews addressing this concept are limited and predominantly recent, further reinforcing the rationale for the present scoping review [12].

Screening

The studies identified from the database search were initially reviewed for duplicates, after which the remaining records were screened against predefined inclusion and exclusion criteria. Duplicate records were identified and removed both within individual databases and following database combination using EndNote 21 reference management software. Automatic duplicate detection was initially performed using author, title, year, and journal matching, followed by manual verification to identify any remaining duplicate entries not detected electronically. Screening was conducted in two stages: an initial review of titles and abstracts, followed by full-text assessment. Studies deemed potentially relevant at the initial stage were progressed to full-text screening.

Inclusion criteria: The inclusion criteria were as follows: peer-reviewed articles, publications in English, studies involving human subjects or human cell lines, studies involving animal subjects or animal cell lines, research articles defining or discussing radiosensitivity and/or radiosusceptibility, studies addressing individual variation in response to ionising radiation, and studies providing guidance or recommendations for individual radiation risk assessment.

Exclusion criteria: The exclusion criteria were as follows: non-peer-reviewed or non-empirical sources (e.g., editorials, opinion pieces), non-English language articles, studies not directly addressing the review question, articles not discussing radiosensitivity and/or radiosusceptibility, secondary sources (e.g., reviews, textbooks), and articles published prior to the year 2000.

Considerations in Study Selection

A key consideration in the screening process was the inclusion of both human and animal studies, as well as in vivo and in vitro research. Radiation effects differ significantly between isolated cells and whole organisms, and further variability exists between species. This variability is well documented [13] and presents challenges when translating findings into clinical practice, particularly in the development of radiation protection guidelines and risk assessment models.

While concerns regarding the direct applicability of animal or in vitro data to human populations were recognised, excluding such studies would have resulted in an incomplete representation of the field. Much of the foundational knowledge in radiobiology has been derived from experimental models, including animal studies and cell line research. Therefore, these studies were retained to ensure a comprehensive overview of the available evidence.

Studies published prior to the year 2000 were excluded from the formal screening process to maintain a contemporary focus aligned with the aims of this scoping review. Although foundational concepts relating to radiosensitivity emerged during the early and mid-20th century, the understanding and application of both radiosensitivity and radiosusceptibility have evolved substantially over the past two decades, particularly following advances in molecular biology, genomics, DNA damage response pathways, radiogenomics, and large-scale epidemiological modelling. Restricting the review to literature published from 2000 onwards, therefore, enabled greater relevance to current scientific understanding, clinical practice, and modern radiological protection frameworks, while also reducing conceptual heterogeneity associated with historical terminology and methodologies.

Refinement of Search Results

Application of the initial search strategy yielded a large volume of results, with a combined dataset exceeding 14,000 records, making direct screening impractical. Preliminary inspection indicated that a substantial proportion of these studies were not directly related to ionising radiation. To address this, further stratification was undertaken using EndNote 21 reference management software. An initial filtering step required the presence of the term “radiation” (or related terms such as “radiotherapy”) within the article title. This approach reduced the dataset to approximately 3,500 records while maintaining relevance to the review topic.

Consideration was given to using more specific terms, such as “radiosensitivity”, within the title as an additional filtering step. However, this approach was found to be overly restrictive, reducing the dataset to approximately 307 records in a single stage and risking the exclusion of potentially relevant studies that may not explicitly use this terminology in their titles.

Additional refinement was achieved by requiring the term “radiosensitivity” to be present within the abstract. This decision was based on the expectation that studies centrally concerned with radiation response would explicitly reference this concept. This step reduced the dataset to approximately 800 records, which were then considered manageable for detailed title-level screening.

The term “radiosusceptibility” was not used as a primary filtering criterion at this stage, as preliminary searches indicated that it yielded very few results. This reflects the relatively limited and emerging use of this term within the literature. Relying solely on this term risked excluding relevant studies addressing similar concepts using alternative terminology.

The staged filtering approach adopted in this review reflects a balance between comprehensiveness and feasibility. While the use of specific terms such as “radiation” in the title and “radiosensitivity” in the abstract may have excluded some relevant studies, it enabled systematic reduction of an otherwise unmanageable dataset. This approach aligns with the overall aim of maintaining a focused yet sufficiently broad exploration of the literature.

Data Extraction

A standardised data extraction form was developed (Table 1) and applied to all included studies to ensure consistency and transparency in data collection. Extracted data were systematically recorded and are presented in a tabulated format.

Findings

Study Characteristics

**Figure 2:**
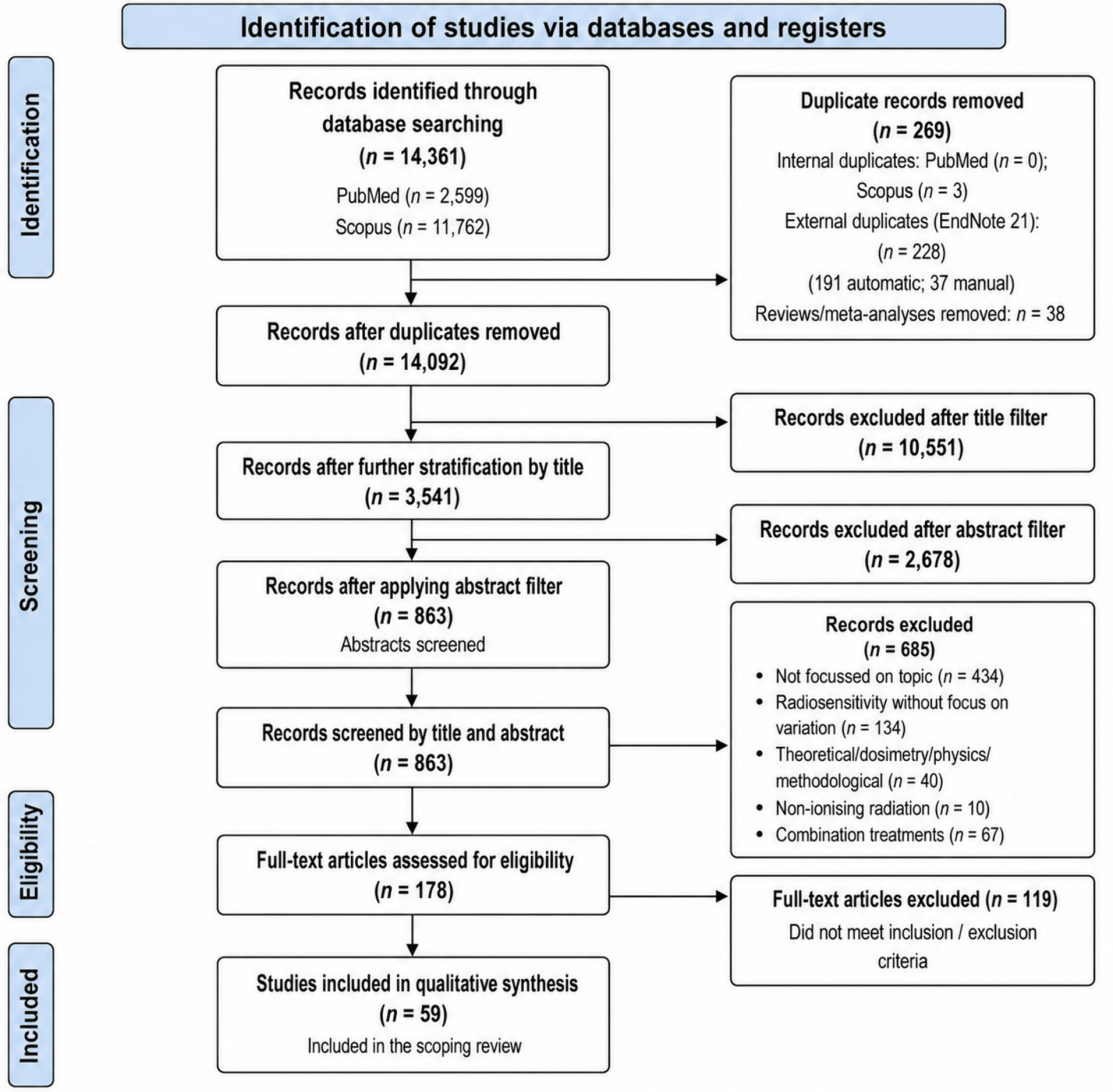
PRISMA flow diagram for study selection Image Credit: Abdul Nadim Asil

The study selection process is summarised in Figure 2. A total of 14,361 articles were initially identified through database searching, comprising 2,599 records from PubMed and 11,762 from Scopus. These records were screened for duplicates. No internal duplicates were identified within the PubMed dataset, while three internal duplicates were identified and removed from the Scopus results.

Following consolidation of the datasets using EndNote 21 reference management software, a further 228 duplicate records were identified and removed, of which 191 were detected automatically and 37 manually.

Although review articles and meta-analyses were specified within the exclusion criteria and corresponding filters were applied during the initial database searches, a small number of such records remained within the exported search results. This occurred because database indexing and publication categorisation are not always applied consistently across journals and databases, resulting in some review and meta-analysis articles not being identified by the available search filters. Following the importation of all records into EndNote 21, an additional screening step was undertaken prior to title and abstract review. During this stage, 38 review and meta-analysis articles were identified through publication type metadata and manual verification and were subsequently removed from the dataset. These exclusions occurred after duplicate removal but before the application of title- and abstract-level screening criteria, resulting in a total of 14,092 records.

Application of the initial search strategy yielded a large volume of results, with a combined dataset exceeding 14,000 records, making direct screening impractical. Preliminary inspection indicated that a substantial proportion of these studies were not directly related to ionising radiation and would therefore be unlikely to contribute meaningfully to the review question. To address this, further stratification was undertaken using EndNote 21 reference management software.

An initial filtering step required the presence of the term “radiation” or a closely related term within the article title. Related terms included: “radiotherapy”, “radiation therapy”, “radiation-induced”, and “radiation response”. This filtering process was conducted electronically using the title-search functionality within EndNote 21 and served as a pragmatic method of removing studies with no obvious relevance to radiation biology or radiation response. Application of this criterion reduced the dataset from 14,092 to 3,541 records while maintaining a broad scope relevant to the review objectives.

Further refinement at the abstract level, through application of key terminology, yielded 863 articles for abstract screening.

**Figure 3:**
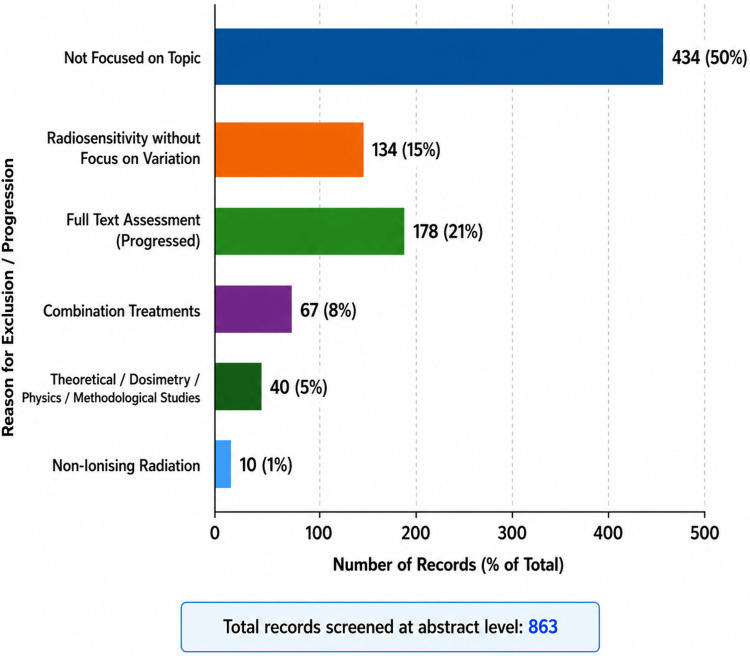
Breakdown of exclusions Distribution of records screened at abstract level by reason for exclusion or progression to full text assessment. Not focussed on topic (50%), theoretical (5%), combination treatments (8%), radiosensitivity without focus on variation (15%), non-ionising radiation (1%), full text assessment (21%). Image Credit: Lily Crouzier

The bar chart illustrates the distribution of the 863 screened articles across the thematic domains identified during the screening process (Figure 3). The largest category comprised studies not directly focused on the review topic (n = 434). These articles were unrelated to radiosensitivity or individual variation in response to ionising radiation and encompassed a broad range of unrelated radiobiological investigations. Examples included studies examining microbial radiation survival mechanisms, such as the screening of genes involved in radiation survival in *Escherichia coli* [14], as well as mechanistic investigations of radiation-induced cell death pathways that did not address variability in radiation response, including studies of apoptotic signalling in human cervical squamous cell carcinoma cells [15]. Although these studies contributed to broader radiobiological understanding, they did not align with the specific objectives of this review.

The second largest category included studies addressing radiosensitivity without specifically examining inter-individual variability or radiosusceptibility (n = 134). These studies generally investigated molecular or therapeutic modifiers of radiation response in experimental systems. For example, some explored the role of microRNAs in altering tumour radiosensitivity, such as miRNA-214-5p targeting ROCK1 expression in cervical cancer [16], while others examined the influence of compounds such as selenium on the radiation sensitivity of glioma cells [17]. Although relevant to radiation response broadly, these studies did not investigate differences between individuals or populations and were therefore excluded from the final synthesis.

Additional excluded studies included those primarily focused on theoretical, dosimetric, or methodological aspects of radiation science (n = 40), studies investigating non-ionising radiation modalities (n = 10), and studies involving combination treatment approaches such as chemotherapy or immunotherapy (n = 67). While some of these articles referenced radiosensitivity, their principal focus fell outside the conceptual scope of this review and were thus excluded from this review.

Of the 863 articles screened at the abstract level, 178 progressed to full-text assessment. Following detailed evaluation, 59 studies were identified as directly addressing radiosensitivity, radiosusceptibility, and/or variation in response to ionising radiation. These studies were included in the final analysis, forming the basis of this review, with their characteristics summarised in Table 2.

**Table 2:** Data extraction table

Study ID	Study Design	Sample Size	Population Characteristics	Population Type	Type of Ionising Radiation	Radiation Dose	Source/Technique	Radiosensitivity Indicators	Radiosusceptibility Indicators	Other Outcomes	Radiosensitivity Testing Methods	Radiosusceptibility Assessment	Radiosensitivity Results	Radiosusceptibility Findings	Significance	Genes of Interest	Molecular Pathways/Biomarkers
Ahmed et al. (2016) [18]	Retrospective clinical observational study	33 patients	Patients with liver metastases from different primary cancers	Human	SBRT	50-60 Gy in 5#	SBRT	Tumour response to radiation	Histology-based variation	Local control rate	Clinical imaging outcomes	Comparison across tumour histology	Differences in response based on tumour origin	Histology influences radiation response	Primary histology may be a factor in radiosensitivity and thus SBRT dose selection	N/A	N/A
Alchinova et al. (2015) [19]	Observational	32	Pilots, flight technicians, parachutists	Human subjects	Environmental/Occupational (cosmic IR) Likely low dose	Not Recorded	N/A	Radioadaptive response as a functional test	Functional evaluation	Functional performance tests	Radioadaptive response test	N/A	The intensity of RAR depends on genetic predisposition and the physiological status of the body	The intensity of RAR depends on genetic predisposition and the physiological status of the body	Potential use of RAR in evaluating the functional performance of pilots	N/A	N/A
Alsbeih et al. (2003) [20]	Case Report	1	Paediatric cancer patient with +FHx of RS, chromosomal fragility syndrome	Human Patient, 3 y/o F	Reduced standard dose RT	19.5 Gy in 13#	Gamma source	Phytohemaglutinin-stimulated peripheral blood lymphocytes showed 9% spontaneous chromosome aberrations	Chromosomal fragility syndrome diagnosis	AT gene sequencing revealed no mutations, recommendations for RT dose modification	Colony formation assay	Based on family history and genetic predisposition	Sensitivity of patients' fibroblasts found to be unusually high, though less sensitive than AT	Use of Family History as a potential indicator	Requirement of tailored radiotherapy dosing	AT Gene	N/A
Alsbeih et al. (2014) [21]	Genetic association study	155 patients	155 head and neck cancer	Human	Standard RT	Standard RT 66Gy with 2Gy per fraction	6MV linear accelerator	Allelic frequency of SNPs	HDM2 polymorphism	Late tissue reactions	Genotyping of SNPs	Not investigated	6 SNPs showed significant association with radiation toxicity.	Not Investigated	SNPs in the promoter region are significantly associated with fibrotic reaction	CDKN1A TP53, ATM, PRKDC, XRCC4 HDM2 XRCC3, XRCC5 XRCC1 LIG4 TGFB1	NHEJ, HR
Arlett et al. (2008) [22]	Lab-based experimental study	33 Patients with XP	Fibroblast cell strains from these patients	Human fibroblast cells, biopsies taken	Co-60 source	5 Gy to 1 Gy min^-1^	Gamma source	Survival fraction post-irradiation	Comparison of XP fibroblasts' sensitivity to non-XP fibroblast / normal human as a control	N/A	Colony formation assay	Comparative analysis	XP fibroblasts exhibited minimal sensitivity to IR	XP fibroblasts were not significantly more susceptible to IR	Challenged the expectation that XP fibroblasts would be highly sensitive to IR	XPC	N/A
Ban et al. (2004) [23]	Experimental In vitro	216 Patients	48 healthy controls, 130 Breast, 31 Cervical, 7 Head + Neck cancer patients, all female	Blood samples - peripheral blood lymphocytes	X ray	0 Gy and 2 Gy, 1 Gymin-1	X-ray generator at room temperature	Micronucleus formation in peripheral blood lymphocytes	Increased micronucleus formation correlated with clinical RS	A small group also had chemotherapy - no indication of association with the type of chemotherapy and in vitro induced MN frequency	Micronucleus assay	Based on the frequency of micronuclei in lymphocytes after irradiation	Breast and cervical patients had significantly different MN frequency, non significantly different for H+N	Higher micronucleus formation in patients with clinical radiosensitivity	MN assay can potentially predict radiosensitivity in cancer patients	N/A	N/A
Barber et al. (2000) [24]	Experimental In vitro	312 patients	All female, post op RT following WLE for breast carcinoma, all primary tumour, no mets/prev malignancy	Blood samples - peripheral blood lymphocytes	X-rays for RT	40 Gy 15# over 20days using 4-8 MV photons	RT treatment, detail of source not provided	Chromosomal aberration in peripheral blood lymphocytes	Clinical tissue reactions, acute and late, fibrosis, breast retraction, telangiectasia	AT heterozygotes have scores for high dose rate MN significantly higher	Micronucleus assay (high dose rate and low dose rate) + Paterson G_2 _assay	Based on the correlation between chromosomal radiosensitivity in vitro and clinical tissue reactions.	Significantly more sensitive to the MN assay with higher-grade tumours	Significant correlation was found between higher chromosomal radiosensitivity and increased normal tissue damage following radiotherapy.	MN assay can potentially predict radiosensitivity in cancer patients	N/A	N/A
Barwell et al. (2007) [25]	Observational	2241 patients	BC post treatment & unaffected controls - 1768, newly diagnosed and UC - 248, newly diagnosed, untreated BC & UC - 225	DNA extract from WBC	X-ray RT	0.5 Gy, 1 Gy, 4 Gy	Caesium 137	Telomere length in peripheral blood lymphocytes - in vitro chromosome radiosensitivity - chromosome breakage using G_2 _assay, induced apoptotic response to irradiation	Not measured	Correlation between telomere length and cancer susceptibility	G2-Assay, Apoptotic assay	Not directly assessed	Did not find a direct correlation between telomere length and radiosensitivity.	No direct findings on radiosusceptibility.	Telomere length was not found to be a significant biomarker for predicting cancer susceptibility or radiosensitivity.	Not directly studied, though genes associated with telomere maintenance are of interest	Telomere biology, Homologous recombination
Bergqvist et al. (2001) [26]	Experimental In vitro	2 Human lung cancer cell lines	SCLC line and NSCLC line	Lung cancer cell lines	X-ray	0, 1, 2, 4, 10, 20 Gy all in 1 fraction	Caesium 137	Frequency of micronuclei formation in cancer cell lines.	Not measured	Correlation between micronuclei frequency and radiation sensitivity.	Micronucleus assay + Colony formation	N/A	A higher frequency of micronuclei was correlated with increased intrinsic radiation sensitivity	Not Investigated	Counting micronuclei is not sensitive enough for the estimation of radiosensitivity in clinical doses. Results demonstrated a distinct difference between NSCLC and SCLC cell lines at higher doses	N/A	DNADR implicated
Brzozowska et al. (2012) [27]	Observational	73 patients	23 Healthy controls, 25 Prostate cancer patients with severe side effects and 25 patients without severe side effects	Lymphocytes from peripheral blood samples	γ-ray	0.5 Gy & 1 Gy	Not specified	Frequency of chromatid aberration	Acute clinical toxicity and late toxicity were measured	Chromatid aberrations are classified as per Sanford et al. (1989)	G_2 _Assay	Chromosomal radiosensitivity in cancer patients compared to healthy donors	Prostate cancer patients have an increased aberration level	Predictive value of the G2assay doubtful for the 25% of patients who showed both severe clinical side effects	Prostate cancer patients have a statistically significant increased aberration level measured by the G2 assay when compared to healthy donors. Although no obvious correlation between clinical and cellular radiosensitivity in lymphocytes of prostate cancer patients when all chosen in vitro assays are considered.	N/A	N/A
Bürger et al. (2006) [28]	Experimental in vitro	33 fibroblast lines	NBS patients and carriers	Human (genetically predisposed)	X-ray	5 Gy	6MV linear accelerator	DNA damage and repair	Genetic disorder (NBS)	DNA repair deficiency	Comet assay	Genetic status	Increased DNA damage	High susceptibility in NBS	Clear biological differences	NBS1	DNA repair pathways
De Ruyck et al. (2005) [29]	Observational genetic study	62 female patients	Cervical or endometrial cancer	Human	Standard RT	Dose not mentioned	Not specified	Late clinical radiosensitivity	DNA repair gene polymorphisms	Cancer incidence	Clinical toxicity assessment	Genotyping	Variability in late toxicity	Association with XRCC genes	Genetic associations identified	XRCC1, XRCC3, XRCC5	DNA repair
Dong et al. (2012) [30]	Case-control Study	517 patients	Chinese Han population, with Gastric Cancer	Human	No Radiation	N/A	N/A	Chromatid breaks per cell	Gastric cancer risk	N/A	Mutagen sensitivity assay	N/A	Higher sensitivity in cases	Strong association with cancer risk	Cases of gastric cancer have higher radiation sensitivity based on chromatid breaks per cell	N/A	N/A
Dulong et al. (2020) [31]	Translational experimental study	16 RT patients	Patients with severe radiotherapy side effects	Human (fibroblast-derived)	X-ray	2 Gy at dose rate 0.8 Gy/min	XRAD320 X-ray generator	Fibroblast response	Clinical toxicity	Gene expression	Cellular assays	Clinical phenotype correlation	Altered sensitivity in affected patients	NFATC2 involvement	Fibroblasts From Patients With Severe Radiotherapy Side Effects Exhibit Decreased Tolerance to Radiation Toxicity	NFATC2	N/A
Dyomina et al. (2007) [32]	Experimental In vitro	103 patients	103 healthy individuals, adults	Lymphocytes from peripheral blood samples with metaphase analysis	X-ray	1.5 Gy	Not specified	Chromosomal aberrations in lymphocytes	Increased frequency of chromosomal aberrations as an indicator of individual radiosensitivity	Potential link between chromosomal radiosensitivity and cancer risk/cancer susceptibility	G_2 _Assay	Based on the frequency of chromosomal aberrations after exposure to radiation	Some (12%) individuals exhibited higher chromosomal radiosensitivity, indicating potential cancer risk	Some suggest that increased chromosomal radiosensitivity in lymphocytes may correlate with a higher risk of developing cancer	Individual chromosomal radiosensitivity could serve as a biomarker for cancer risk assessment	N/A	DNADR implicated
Girard et al. (2020) [33]	Experimental In vitro	5 Human cell lines	Chondrosarcoma cell lines with different genetic backgrounds and radiation responses	Chondrosarcoma cell lines cultured in normoxia or hypoxia, sensitivities determined by survival curves	X-ray + hadrontherapy with Carbon ions	Doses ranging from 2 to 8 Gy were tested for both types of radiation.	X-ray irradiator and carbon ion accelerator	Cell viability and clonogenic survival after irradiation	Differences in survival rates and DNA damage responses among the cell lines.	Comparison of the effectiveness of X-rays versus carbon ions in inducing cell death	Colony formation assay	Based on the survival fraction and DNA damage response after irradiation	X-ray radiations induced death in some chondrosarcomas by both apoptosis and senescenceFound heterogeneity in the response of chondrosarcoma cell lines to both X-rays and carbon ions, with some lines being more sensitive to carbon ions	Carbon ions were generally more effective in reducing cell survival, but responses varied among cell lines	Carbon ion therapy could be more effective for certain chondrosarcomas, but patient-specific factors must be considered	TBX3, CDK2A, HMGA2, p21 Expression	DNA damage monitored by γH2AX expression. Apoptosis was assessed by cell cycle analysis and Apo2.7 expression and evaluating PARP cleavage. Senescence evaluated using SA β-galactosidase assay. Necrosis and autophagy were evaluated by RIP1 and beclin-1 expression, respectively
Golomb et al. (2024) [34]	Observational comparative study	81 patients	Veterans with gulf war illness (n = 41) vs civilians (n = 40)	Human	Environmental: X-ray, radioactive chemicals,	Not specified	Not specified	Adverse effects	Illness status	Symptom burden	Clinical assessment	Group comparison	Increased adverse effects	Higher susceptibility in the affected group	Greater RadAE vulnerability fits an emerging picture of heightened drug/chemical susceptibility in VGWI.	N/A	N/A
Guo et al. (2004) [35]	Experimental In vitro	18 patients	Patients with cerebral AVM post op from Gamma Knfie radiosurgery for AVM adults	Lymphocytes from peripheral blood samples	External Gamma irradiation	8 Gy	Not specified	DNA DSB in 50 randomly selected individual nuclei	Differences in the number of DSB measured at 1 hr and 2 hr post irradiation	Nil recorded	Neutral single-cell gel electrophoresis/comet analysis	Not directly assessed.	Individuals with less post-therapeutic regression in AMV sizes or relatively poor or inadequate responses to radiosurgery were shown to have significantly higher DSB repair capacity on their leukocytes by comet analysis	Not Investigated	These results suggested that in vitro radiosensitivity of peripheral leukocytes may provide valuable information for predicting therapeutic response or for adjusting irradiation doses in AVM radiosurgery	N/A	N/A
Hamasaki et al. (2007) [36]	Experimental In vitro	6 patients	6 healthy adults aged between 33 and 49	Lymphocytes from peripheral blood samples	X-ray	1, 2, 4, 8 Gy at 0.7 Gy min^-1^	X-ray irradiator	γH2AX foci formation as an indicator of DNA double-strand breaks	Variation in γH2AX foci formation among individuals, indicating different levels of DNA damage response	Assessed the correlation between γH2AX foci formation and individual radiosensitivity	γH2AX flow cytometry and short-term culture of lymphocytes	Based on the quantity of γH2AX foci as a marker of DNA damage and repair capacity	Found significant inter-individual variability in γH2AX foci formation, indicating differences in radiosensitivity	Individuals with higher levels of γH2AX foci formation were more radiosensitive, suggesting that γH2AX is a potential biomarker for radiosensitivity	γH2AX flow cytometry could be used as a rapid and sensitive method for assessing individual radiosensitivity	DNADR related to γH2AX	DNA damage response pathway
Haston et al. (2002) [37]	Experimental genetic animal study	94 Mice	Mice strains	Animal model	Not specified	14 or 16 Gy depending on strain	Not specified	Pulmonary fibrosis development	Genetic loci influencing susceptibility	Fibrosis severity	Phenotypic assessment post-irradiation	Genetic linkage analysis	Variation in fibrosis response	Identification of susceptibility loci	Genetic determinants identified	Not specified (loci identified)	N/A
Howe et al. (2009) [38]	Experimental In vitro	80 patients	20 healthy controls, 60 colorectal carcinoma patients, adults	Lymphocytes from peripheral blood samples	Co-60 source	0.5Gy	Cobalt 60 source	Chromosomal aberrations in lymphocytes	Bystander effects, including DNA damage in non-irradiated cells adjacent to irradiated ones	Examination of the relationship between intrinsic radiosensitivity and bystander effects	G_2_ assay + cell viability testing	Extent of bystander effects observed in non-irradiated cells	No direct correlation between intrinsic radiosensitivity and the extent of bystander effects	Bystander effects were observed across different radiosensitivity profiles, suggesting they are independent phenomena	The study suggests that bystander effects may have clinical significance, but they are not directly related to an individual's intrinsic radiosensitivity	N/A	N/A
Jost et al. (2022) [39]	Experimental In vitro (Open cohort)	49 patients	Adults with cancer treated with a kinase inhibitor	Lymphocytes from peripheral blood samples	X-ray	2 Gy	X-ray irradiator/linear accelerator	Cell viability and DNA damage (e.g., γH2AX foci formation) post-irradiation	Increase in radiation sensitivity when cells are treated with kinase inhibitors	Analysis of how kinase inhibitors modulate the radiosensitivity of normal cells in cancer patients	Colony formation assay + γH2AX staining to measure DNA damage + FISH to measure individual RS via chromosomal aberrations	Based on changes in cell survival and DNA damage when kinase inhibitors are applied before irradiation	Kinase inhibitors were found to increase the radiosensitivity of normal cells in cancer patients	Kinase inhibitors could exacerbate radiation-induced damage in normal tissues, leading to increased radiosensitivity	Kinase inhibitors might require careful management during radiotherapy to avoid excessive damage to normal tissues	Not specified	Pathways affected by kinase inhibitors, including those regulating DNA repair and cell survival
Kelsey et al. (2013) [40]	Clinical genetic study	39 patients	Lung cancer patients	Human	Conventional RT	40-70 Gy (fractionated in 1.8-2 Gy fractions ± concomitant boost)	Not specified	Lung toxicity	SNPs	Genetic polymorphisms in genes involved in DNA damage repair. XRCC1 and BRCA1	Clinical and radiological endpoint	Genotype frequencies	Variation in lung response	Genetic associations identified	Dosimetric factors predict the risk of radiation-induced lung injury but have limitations. Underlying genetic factors, including SNPs, may contribute to risk.	Multiple SNPs	N/A
Kitahara et al. (2002) [41]	Experimental In vitro	19 patients	19 adults with cervical cell carcinoma	Cervical squamous cell carcinoma tissues	X-ray plus Ir 192 high-dose intracavity brachytherapy	30.6 Gy + 52.2Gy	X ray irradiator + Iradium 192	Gene expression profiles associated with radiosensitivity or resistance	Expression levels of specific genes correlated with either increased sensitivity or resistance to radiation	Classification of cervical cancer samples based on their gene expression profiles	cDNA microarray analysis to identify gene expression patterns	Based on the correlation between gene expression profiles and clinical outcomes post-radiotherapy	Identified 62 genes whose expression profiles could classify cervical cancers as either sensitive or resistant to ionising radiation	Specific gene expression profiles were linked to either radiosensitivity or radioresistance in cervical cancer	Gene expression profiling could help predict the response of cervical cancers to radiotherapy, potentially guiding treatment decisions	62 genes identified, including those involved in DNA repair, cell cycle regulation, and apoptosis. To name a few: XRCC5, ALDH1, MAP3K2, RAB5C	Pathways relating to DNA repair, cell cycle control and apoptosis
Kumar et al. (2014) [42]	Experimental In vitro	4 different cell lines	Raji cells (B lymphoblastoid cell line / Burkitt lymphoma), MCF-7 (human breast cancer cells), A431 cells (epidermoid carcinoma), U937 cells (human histiocytic lymphoma cells)	In vitro on various human tumour cell lines	Beta radiation from Iodine-131 (I-131) and gamma radiation	0.4Gy and equivalent dose from a gamma source	I-131 as the source of beta radiation, and a Co-60 for gamma radiation	Cellular viability, apoptosis, and DNA damage after radiation exposure	Differences in the extent of apoptosis and DNA damage between beta and gamma radiation	Comparative analysis of the cellular and molecular effects of beta and gamma radiation on tumour cells	Cell viability assays (trypan blue assay), flow cytometry for apoptosis, and comet assay for DNA damage	Based on the differential responses of tumour cells to beta versus gamma radiation	Beta radiation (I-131) was found to be more effective in inducing DNA damage and apoptosis compared to gamma radiation	Beta radiation may be more effective in targeting certain tumour cells due to its higher linear energy transfer (LET)	I-131 beta radiation could be a more potent therapeutic option compared to gamma radiation for specific tumours	Not specified	RAD51 & p21 expression
Lanvin et al. (2013) [43]	Experimental In vitro	1 type of cell line	U87 Glioblastoma cell line	Glioblastoma tumour cells	X-ray	2 Gy	X-ray irradiator	Mitotic cell death, DNA damage response, and cell survival post-irradiation	Role of integrin-linked kinase (ILK), hypoxia-inducible factor 1alpha (HIF-1α), and survivin in regulating radiation response	Identification of pathways contributing to radioresistance in glioblastoma	Cell viability assays, flow cytometry for apoptosis and mitotic death, Western blotting for protein expression	Based on the modulation of ILK, HIF-1α, and survivin, which influence radiation resistance	Increased expression of ILK, HIF-1α, and survivin contributes to radioresistance by promoting mitotic cell death	Targeting these pathways could enhance radiosensitivity in glioblastoma, potentially improving therapeutic outcomes	Understanding the molecular mechanisms of radioresistance could lead to more effective radiotherapy strategies for glioblastoma	ILK, HIF-1α, Survivin	Pathways involving ILK, HIF-1α, survivin, and mitotic cell death regulation
Lee et al. (2008) [44]	Experimental in vitro	2 types of head and neck cancer cell line study	AMC-HN-3 and -9 cell lines	Human head and neck cancer cell lines	X-ray	4 Gy	6MV linear accelerator	Protein expression differences	Proteomic variation	Other proteins identified in the Western blot	Protein identification via MALDI TOF Mass Spec	Not specified	Differences in protein expression linked to sensitivity	Molecular differences identified	Expression of different proteins may perform important functions regarding radio resistance	N/A	N/A
Lemke et al. (2014) [45]	Experimental In vitro	Glioblastoma cell line	Primary cultures derived from multiple unspecified amount of adult patients	Glioblastoma initiating cells	X-ray	4 Gy and 8 Gy	X-ray irradiator	Cell viability, clonogenic survival, and expression of stem cell markers post-irradiation	Correlation between stem cell marker expression and radiotherapy sensitivity	Exploration of whether profiling stem cell markers can predict radiosensitivity in glioblastoma	Colony formation assay, flow cytometry for stem cell markers, gene expression analysis	Expression levels of stem cell markers and their correlation with radiation response	Certain stem cell markers (e.g., CD133) are associated with increased radioresistance in glioblastoma	Glioblastoma cultures with higher expression of stem cell markers were more resistant to radiotherapy, suggesting that these markers could predict treatment outcomes	Targeting stem cell populations within glioblastomas could improve the effectiveness of radiotherapy	CD133 and other stem cell markers	Associated with CD133 and stem cell maintenance
Li et al. (2014) [46]	Experimental In vitro/In vivo	Mice tumour cell lines	Two cell lines isogenic, DNA-PKcs defective line and a competent DNA-PKcs repair gene line	In vitro cultured cells transferred to hind leg of 8-9 wk old male mice	X-ray	5 Gy and 11.2 Gy	X-ray irradiator, 320kVp @ 1.66 Gy min^-1^ and 3.76 Gy min^-1^ for 3 mins	Tumour cell survival, stromal response, and achievement of permanent local control	Interaction between tumour cells and stroma, with emphasis on how stromal response affects overall tumour radiosensitivity	Analysis of the role of tumour stroma in influencing the overall response to radiotherapy	Tumour growth delay assays, colony formation assay, and histological analysis of stroma and tumour interactions	Combined effects of radiation on both tumour cells and tumour stroma, affecting overall treatment outcomes	Both tumour cell radiosensitivity and stromal response are critical for determining the effectiveness of radiotherapy	Tumour stroma plays a significant role in determining radiosensitivity, and its response can either enhance or mitigate tumour control	Effective radiotherapy requires considering both tumour cell intrinsic radiosensitivity and the modifying effects of the tumour microenvironment	DNA-PKcs repair gene for DNA damage	DNA-PKcs and stromal signalling pathway
Liu et al. (2006) [47]	Experimental In vitro	Human cancer cell lines	C4-1 (HPV 18+), HeLa (HPV 18+), SiHa (HPV 16+), and Caski (HPV 16+)	4 cervical cancer cell lines	γ-ray	2 Gy and 6 Gy	MDS Gammacell 3000	Cell viability, apoptosis, and clonogenic survival post-irradiation	Overexpression of p73α and its impact on radiation response	Potential of p73α as a target to enhance radiosensitivity in cervical cancer	Colony formation assay + flow cytometry for apoptosis, and Western blotting for protein expression	Based on the degree of enhanced radiosensitivity observed in cells overexpressing p73α	Overexpression of p73α significantly enhanced the radiosensitivity of cervical cancer cells	p73α promotes apoptosis in response to radiation, contributing to increased cell death and radiosensitivity	p73α could be a potential therapeutic target for sensitising cervical cancer to radiotherapy	p73α	Apoptosis pathways regulated by p73α
Lu et al. (2006) [48]	Experimental In vitro	Mice tumour cell lines	25 mice: 17 Atm+/− and 8 wild-type	Mouse model: Atm-haploinsufficiency and WT controls	X-ray	1.1 Gy	Caesium 137	Incidence of mammary tumours in response to carcinogens	Influence of Atm-haploinsufficiency on tumour susceptibility	Atm's role in protecting against carcinogen-induced tumorigenesis	Observation of tumour development in response to carcinogen exposure	Based on the increased susceptibility to tumours in Atm-haploinsufficient mice	Atm-haploinsufficient mice exhibited a higher incidence of mammary tumours, indicating increased susceptibility	Haploinsufficiency of Atm enhances vulnerability to tumorigenesis, possibly due to impaired DNA repair	Atm gene dosage is critical for protecting against carcinogen-induced tumours, highlighting the importance of DNA repair pathways in cancer prevention	Atm (Ataxia Telangiectasia Mutated)	DNA DR involving Atm - genomic stability regulators
Almodóvar et al. (2002) [49]	Experimental In vitro	226 samples of lymphocytes	226 samples of women with breast cancer	Lymphocytes from a peripheral blood sample	X-ray	Therapeutic doses used in standard radiotherapy protocols for breast cancer	Conventional radiotherapy equipment	DNA damage levels in normal cells, assessed before radiotherapy. Quantified as the initial number of DNA double-strand breaks (dsb) induced per Gy and per DNA unit (200 Mbp)	Correlation between DNA damage levels and normal tissue reactions post-radiotherapy	Exploration of the potential to individualise radiotherapy based on DNA damage responses in normal cells	DNA damage assay (Comet assay) performed on normal cells before radiation treatment	Based on the levels of DNA damage in normal cells and their correlation with clinical radiosensitivity (e.g., skin reactions)	Patients with higher DNA damage in normal cells exhibited more severe normal tissue reactions during radiotherapy	DNA damage assays could potentially predict individual radiosensitivity and guide personalised radiotherapy	Assessing DNA damage in normal cells before radiotherapy could help tailor treatment plans to minimise side effects	N/A	N/A
Mayo et al. (2019) [50]	Experimental In vitro	38 patients with 244 healthy controls	38 adults with lung cancer	Lymphocytes from a peripheral blood sample	X-ray	Therapeutic doses used in standard radiotherapy protocols in lung cancer	Conventional radiotherapy equipment	Chromosomal aberrations in G0 phase lymphocytes assessed by three-colour fluorescence in situ hybridisation (FISH)	Frequency of chromosomal aberrations and their correlation with clinical radiosensitivity	Identification of patients with higher radiosensitivity to tailor radiotherapy	G0 phase chromosomal aberration analysis using FISH	Based on the frequency of chromosomal aberrations and their correlation with adverse effects post-radiotherapy	Patients with a higher frequency of chromosomal aberrations showed increased radiosensitivity, manifesting as more severe normal tissue reactions	The study supports using FISH-based chromosomal analysis to predict individual radiosensitivity	FISH-based chromosomal aberration analysis could be a valuable tool for personalising radiotherapy in lung cancer patients	N/A	Chromosomal stability as a biomarker for radiosensitivity
Meehan et al. (2021) [51]	Biomarker Discovery Study	Human cancer cell line	MCF-7 BC ER+ cell line and ZR-751 cell line	Human breast cancer cell lines	X-ray	2 Gy +0.5 Gy incremental + 5 Gy after development of radioresistance	Faxitron cabinet X-ray system	Discovery of novel biomarkers associated with radiotherapy response	Expression levels of candidate biomarkers and their correlation with treatment outcomes	Identification of biomarkers that could predict radiotherapy response in breast cancer patients	High-throughput screening of biomarkers, followed by validation in clinical samples	Expression levels of identified biomarkers and their correlation with clinical radiosensitivity and treatment outcomes	33 potential biomarkers	Biomarkers identified in the study could help stratify patients based on their likely response to radiotherapy	Contributes to the development of personalised radiotherapy in breast cancer by identifying potential predictive biomarkers	5 proteins (YBX3, EIF4EBP2, DKK1, GNPNAT1 and TK1) had higher expression levels in the radiosensitive cells	DKK1 and GNPNAT1 have the potential to predict sensitivity to RT
Milenkova et al. (2013) [52]	Experimental In vitro	36 patients, 24 female and 12 male, + 20 healthy adults	36 patients with Multiple sclerosis, 18 RR (relapsing remitting) and 18 secondary progressive	Multiple sclerosis patients	γ-ray	1.5 Gy	Co-60 source	Frequency of chromosomal aberrations in lymphocytes	Increased chromosomal aberrations in MS patients compared to controls	MS patients exhibit higher chromosomal radiosensitivity, which could have implications for their response to environmental or therapeutic radiation exposure	Chromosomal aberration analysis in lymphocytes exposed to radiation	Based on the frequency of chromosomal aberrations in lymphocytes post-irradiation	MS patients showed higher chromosomal radiosensitivity than healthy controls	Increased chromosomal radiosensitivity in MS patients suggests a possible link between MS and a predisposition to DNA damage	Understanding the aetiology of MS and the management of radiation exposure in these patients	N/A	N/A
Mitsuhashi et al. (2009) [53]	Observational biomarker study	21 patients	Primary, invasive breast cancer	Human	X-ray	0-10 Gy	Cs 137	CDKN1A (p21) mRNA expression	Cancer susceptibility	Biomarker expression differences	Gene expression analysis	Comparison between patient groups	Increased CDKN1A expression post-radiation	Higher expression linked to susceptibility	Suggested biomarker potential	CDKN1A	Cell cycle regulation
Mirzayans et al. (2005) [54]	Experimental In vitro	7 separate cancer cell lines	Normal skin fibroblasts, colon carcinoma, Renal carcinoma, Malignant melanoma, malignant glioma, neuroblastoma	Human solid tumour-derived cell lines	γ-ray	<8 Gy	Co-60 source	Induction of accelerated senescence in response to gamma radiation	Presence of wild-type TP53 and its role in mediating radiation-induced senescence	Relationship between TP53 status and radiation-induced cellular senescence, which is a key determinant of cancer cell radiosensitivity	Assessment of senescence-associated β-galactosidase (SA-β-gal) activity and other markers of senescence post-irradiation	Induction of senescence in response to radiation in cells with wild-type TP53	Cells expressing wild-type TP53 were more likely to undergo accelerated senescence following gamma irradiation	Wild-type TP53 plays a critical role in determining the radiosensitivity of tumour cells via the induction of senescence	Understanding the role of TP53 in radiation-induced senescence could help predict the radiosensitivity of solid tumours and guide therapeutic strategies	TP53	TP53-mediated pathway leading to senescence
Müller et al. (2001) [55]	Experimental In vitro	24 healthy adults, 30 cancer patients	Nasopharynx, oropharynx, tonsil, hypopharynx, oesophagus, lung, breast, cervix and prostate tumour cells	Peripheral blood lymphocytes	X-ray	0.25-2 Gy	Stabilipan X-ray machine	DNA damage levels in lymphocytes as measured by the Comet assay	Increased DNA damage in lymphocytes from cancer patients compared to healthy controls	Aims to explore the potential of the Comet assay as a predictive tool for individual radiosensitivity in cancer patients	Comet assay (single-cell gel electrophoresis) to measure DNA strand breaks in lymphocytes post-irradiation	Based on the degree of DNA damage in lymphocytes post-irradiation	Cancer patients exhibited higher levels of radiation-induced DNA damage compared to healthy individuals	Cancer patients may have an inherently higher radiosensitivity, which could influence their treatment outcomes	Supports the use of the Comet assay in assessing individual radiosensitivity	N/A	N/A
Nakajima et al. (2015) [56]	Experimental in vitro and in vivo	Small cell carcinoma of the uterine cervix in 6 adult human patients	Cancer tissue originated from spheroids developed into a xenograft to 4-week-old female mice	CTOS method for xenograft in vivo studies + Human cancer cells organised into 3D spheroids	X-ray	Dose rate of 0.25 Gy/min for in vitro studies, 5 Gy at a dose rate of 0.1 Gy/min for in vivo	MBR-1505R irradiator	Spheroid viability, growth inhibition, and apoptosis post-irradiation	Variation in radiation response among different spheroids derived from different patients	Potential of using patient-derived spheroids to predict individual responses to radiotherapy	Viability assays, growth inhibition assays, and apoptosis assays were performed on spheroids post-irradiation	Based on the variation in radiosensitivity among spheroids derived from different patients	Spheroids showed heterogeneous responses to radiation, reflecting the variability in radiosensitivity among patients	The findings highlight the potential for using patient-derived spheroids as a model for predicting individual responses to radiotherapy	Irradiation experiments showed variations in radiation sensitivity, both in vitro and in vivo. Elevated levels of HIF-1α were detected in radio-resistant CTOSs within hours of irradiation	HIF-1α	Inhibitor of heat shock protein 90 (HSP90) and related pathways
Pajic et al. (2015) [57]	Experimental In vitro/Comparative	14 healthy individuals	Peripheral blood lymphocytes from healthy donors	Human peripheral blood lymphocytes	Gamma radiation	2 Gy	CLINAC 600EX machine	Frequency of dicentric chromosomes and micronuclei formation in lymphocytes post-irradiation	Variability in the number of dicentric chromosomes and micronuclei between individuals	The study compared two different assays (dicentric and micronucleus) to assess inter-individual variability in radiosensitivity	Dicentric chromosome assay and micronucleus assay	Based on the frequency of dicentric chromosomes and micronuclei as markers of chromosomal damage	Significant inter-individual variability observed in both assays	The dicentric assay and micronucleus assay provided consistent results regarding individual radiosensitivity	The findings emphasise the importance of considering individual variability in radiation response for personalised radiotherapy	N/A	N/A
Peng et al., 2012 [58]	Review component + Experimental In vitro	Human cell lines	Low passage human fibroblast cultures and GM03491 (Atm ^+/+^)	Human fibroblasts	Gamma radiation	0.4, 0.8, 1.2Gy	Caesium 137	DNA damage, chromosomal aberrations	Genetic factors influencing susceptibility to radiation-induced damage	Implications of genetic susceptibility on radiation protection strategies	Chromosomal aberration assays, DNA repair assays	Based on genetic predisposition and observed biological effects in response to radiation	Significant variability in radiation response based on genetic factors	Genetic predisposition plays a crucial role in individual susceptibility to radiation	Understanding genetic susceptibility is key to developing protective measures for astronauts	TP53, ATM, BRCA1/2, among others	DNA repair pathways, cell cycle regulation
Pietrowska et al. (2011) [59]	Experimental In vitro	55 cancer patient adults	46 Male, 9 female, 24 laryngeal, 15 pharyngeal, 16 oral cavity (SCC)	Peripheral blood lymphocytes from these patients	X-ray	Standard RT 52 Gy-76 Gy in 5-7 weeks for patients + 2 Gy in vitro	Linear accelerator (6 MeV energy Clinac 600)	Acute mucosal reaction and plasma proteome profiles	Variations in proteomic profiles and DNA repair capacity	Investigated the relationship between plasma proteome profiles, DNA repair capacity, and acute mucosal toxicity	Mass spectrometry for proteomic analysis, DNA break repair assay in lymphocytes	Based on the correlation between proteome profiles, DNA repair capacity, and clinical outcomes	Specific proteome profiles and lower DNA repair capacity were associated with severe acute mucosal reactions	Plasma proteome profiling and DNA repair assays could be predictive of radiosensitivity	Biomarkers to be used to mitigate and monitor side effects	N/A	Inflammatory response proteins
Pinar et al. (2007) [60]	Experimental In vitro + Normal tissue reactions (Longitudinal study)	40 cancer patients	Locally advanced breast cancer, 39 Female, 1 Male	Peripheral blood lymphocytes from these patients	Gamma radiation	5-45 Gy for in vitro blood samples + standard RT regimes for breast cancer treatment	Co-60 source	DNA damage assessed by γ-H2AX foci formation and long-term toxicity	Extent of γ-H2AX foci formation post-irradiation	γ-H2AX foci as a predictor of long-term radiation-induced toxicity	γ-H2AX foci assay in peripheral blood lymphocytes	Based on the correlation between γ-H2AX foci and long-term side effects	Higher levels of γ-H2AX foci were predictive of long-term radiation toxicity	Supports the use of γ-H2AX foci as a biomarker for predicting radiosensitivity and long-term side effects	Tailoring radiotherapy to reduce long-term toxicity via the use of γ-H2AX	N/A	DNA damage response (γ-H2AX)
Popanda et al. (2003) [61]	Clinical observational study	113 breast cancer patients	Breast cancer patients receiving RT post-breast conserving surgery	Cryo-preserved lymphocytes irradiated and analysed using the comet assay	Gamma radiation	Pt received standard RT, and lymphocytes received 5 Gy in vitro	4-8 MV photon beam	DNA damage and repair capacity	Acute skin reactions	Correlation between biomarkers and toxicity	Lymphocyte assays	Clinical skin toxicity scoring	Variability in DNA repair capacity	Correlation with acute skin reactions	Association between biomarkers and toxicity	N/A	N/A
Reuther et al. (2015) [62]	Partly retrospective, Genetic association study	123 patients	Breast and prostate cancer patients	Peripheral blood lymphocytes from these patients	X-ray	6 Gy	X-ray irradiator	Single-nucleotide polymorphisms (SNPs) associated with early and late radiation toxicity	Specific SNPs linked to increased risk of radiation-induced toxicity	Association between genetic variations and the risk of early or late radiation-induced side effects	Genotyping of SNPs in pathways related to DNA repair, inflammation, and cell cycle regulation + G01 assay	Presence of specific SNPs and their correlation with clinical toxicity outcomes	Certain SNPs were significantly associated with increased risk of early or late radiation toxicity	The findings suggest that genetic profiling could help identify patients at higher risk of radiation-induced side effects	Functional pathways of SNPs may be used to form a risk score allowing to predict acute and late radiation-induced toxicity	N/A	Pathways involving DNA repair, inflammation, and cellular responses to radiation, ROS pathway, TGFB1 signalling
Rosenberger et al. (2012) [63]	Genetic epidemiology study	798 individuals/177 index-persons	Lung cancer patients and their families	Human	Gamma radiation	4 Gy	Not specified	Radiation response assays	Heritability	None	Comet assay from blood lymphocytes	Not specified	Familial variation observed	Significant heritable component	Role of genetic inheritance with a significant heritable component	N/A	N/A
Roy et al. (2006) [64]	Experimental In vitro	3 human glioma cell lines	U87, T98G, U118 glioma cell lines	Glioma cell lines	Gamma radiation	2 Gy, 4 Gy, 6 Gy	Gamma irradiator	Survival fraction post-irradiation, DNA damage, and apoptosis rates	Methylation status of the ATM promoter	Investigation into the correlation between ATM promoter methylation and radiation sensitivity in glioma cells	Colony formation assay, DNA damage assessment, and apoptosis assays	Based on the methylation of the ATM promoter and its impact on radiation response	Glioma cells with methylated ATM promoters exhibited increased radiosensitivity, with reduced DNA repair and higher apoptosis rates	Methylation of the ATM promoter is associated with altered radiosensitivity in glioma cells, suggesting a potential epigenetic mechanism of radiosusceptibility	Epigenetic changes in modulating tumour cell response to radiation, with implications for targeted radiotherapy	ATM	DNA damage response and repair pathways, epigenetic regulation via promoter methylation
Schmitz et al. (2007) [65]	Heritability/Genetic Study	334 healthy individuals	Quiescent lymphocytes from participants (18-93 year olds) 177 F 157M, 325 related from 38 large families	Peripheral blood lymphocytes from these patients	Gamma radiation	0.5, 1, 2 Gy	Cs 137	Apoptosis rates in lymphocyte subpopulations post-irradiation	Heritability of radiation-induced apoptosis susceptibility in lymphocytes	Focused on the genetic basis of susceptibility to radiation-induced apoptosis across different lymphocyte subpopulations	Flow cytometry to measure apoptosis in CD4+, CD8+, and B cell populations	Heritability was assessed by analysing apoptosis rates across family members and determining genetic correlations	Significant variability in apoptosis rates was observed between individuals, with heritable factors contributing to radiosensitivity	Strong heritable component in the susceptibility to radiation-induced apoptosis, particularly in CD8+ T cells	Genetic predisposition plays a critical role in individual radiosensitivity. The segregation of the T4-effector memory radiosensitivity phenotype was consistent with a simple Mendelian transmission model involving one major gene	N/A	Apoptosis pathways, particularly in lymphocyte subpopulations
Seo et al. (2020) [66]	Observational genomic study	59 human solid cancer cell lines	Cancer patients with cell lines derived from 11 organ sites	Human	Gamma radiation	0-12 Gy	Cs 137	Treatment response	Genetic alterations	Mutation burden	Genomic profiling	Mutation analysis	Variation in response	Linked to gene alterations	Significant association between genome-wide aberrations and radiation responsiveness	N/A	N/A
Shahidi et al. (2010) [67]	Comparative study	30 Cancer patients vs 30 healthy controls	Prostate cancer patients aged 45-78 in Iran	Mononuclear cells from these patients	Gamma radiation	0.5 Gy to 16 Gy	Co-60 source	DNA damage and repair kinetics post-irradiation ratio of Tail DNA/Head DNA	Differences in DNA repair capacity between prostate cancer patients and healthy individuals	Compare the radiosensitivity and DNA repair kinetics of leukocytes from prostate cancer patients versus healthy individuals	Alkaline comet assay to assess DNA strand breaks and repair kinetics	Comparison of DNA damage repair rates between the two groups	Leukocytes from prostate cancer patients showed slower DNA repair kinetics compared to healthy controls, indicating higher radiosensitivity	Individuals with sporadic prostate cancer may have an inherent radiosensitivity due to impaired DNA repair mechanisms	Potential for using DNA repair kinetics as a biomarker for radiosensitivity in prostate cancer patients, which could inform treatment strategies	N/A	N/A
Smirnov et al. (2012) [68]	Genome-wide association study	99 healthy individuals	B cell lymphocytes	Peripheral blood lymphocytes	Gamma radiation	10 Gy	Cs 137	Radiation-induced cell death, caspase 3/7 levels and cytotoxicity	Genetic variations associated with differential radiation-induced cell death	Identified single-nucleotide polymorphisms (SNPs) linked to radiosensitivity	High-throughput screening for cell viability post-irradiation	Correlation between SNPs and radiation-induced cell death	Variability in cell death responses among cell lines was associated with specific genetic variants	Identified several SNPs significantly associated with radiosensitivity, indicating a genetic basis for radiation response	Results uncovered DNA variants that contribute to radiosensitivity and identified genes that can be targeted to increase the sensitivity of tumours to radiation	Several found	DNA damage response pathways, apoptosis, and cell cycle regulation
Steffen et al. (2005) [69]	Experimental In vitro	3 Breast cancer cell lines	SKOV-3, SKBR-3 and BT-474, overexpressing HER2	Human Breast cancer cells	Gamma radiation	2-8 Gy	Cs 137	Clonogenic survival and affibody molecules	Differences in radiosensitivity among the three cell lines	Relationship between HER2 overexpression and radiosensitivity	Clonogenic assay, γH2AX foci formation for DNA damage, and apoptosis assays	Comparison of radiosensitivity among the three HER2-overexpressing cell lines	Significant differences in radiosensitivity among the cell lines, with SKBR3 being the most radiosensitive	HER2 overexpression alone did not uniformly increase radiosensitivity; other factors, such as DNA repair capacity, may play a role	HER2 overexpression is not the sole determinant of radiosensitivity in breast cancer cells	HER2	HER2 signalling pathway
Suzuki et al. (2003) [70]	Experimental imaging study	3 human cancer cell lines xenografted to athymic mice	Human tumour xenografts	Animal model	X-ray	Depending on the cell line, 10-20 Gy	4 MV linear accelerator	Tumour response via 99Tc uptake	Not directly assessed	Nil recorded	Nuclear imaging	N/A	Imaging reflects tumour response	N/A	Imaging utility	N/A	N/A
Taghavi-Dehaghani et al. (2005) [71]	Experimental In vitro	26 patients	26 breast cancer patients with early or late tissue reactions	Peripheral blood lymphocytes	Gamma radiation	4 Gy	Co-60 source	Micronuclei formation in lymphocytes, apoptosis rates	Differences in micronuclei formation and apoptosis between pre- and post-radiotherapy samples	Correlation between radiosensitivity (micronuclei) and apoptosis in lymphocytes	Micronucleus assay, apoptosis assay	Comparing radiation-induced micronuclei and apoptosis in lymphocytes before and after radiotherapy	Higher micronuclei formation was observed post-radiotherapy, indicating increased radiosensitivity	Significant differences in the induction of micronuclei and apoptosis, with both being higher in post-radiotherapy samples	Micronuclei and apoptosis can serve as biomarkers for assessing radiosensitivity in breast cancer patients	N/A	Micronuclei formation and apoptosis as indicators of DNA damage and cell death
Tsougos et al. (2005) [72]	Modelling study using clinical data	150 patients	Breast cancer patients	Human	Standard RT	50Gy + modelled dose response	6MV Linear Accelerator	Pneumonitis	Dose-response variability	Model performance	Clinical outcome modelling	Statistical modelling	Predictive models identified	Variability partly dose-driven	Model-dependent	N/A	N/A
Van Eerde et al. (2001) [73]	Experimental animal study	3 different rat strains	Rats receiving hemithoracic radiation	Rats - albino Wistar, Sprague-Dawley, Fisher rats	X-rays	12 or 18 Gy	Not specified	Development of lung injury	Strain-dependent susceptibility	Pulmonary damage severity	Observation of lung injury post-irradiation	Comparative strain analysis	Different rat strains show varying lung injury responses	Genetic background influences susceptibility	Supports the notion that interpatient variability in radiation-induced pulmonary dysfunction is, at least in part, genetically controlled	N/A	N/A
Xu et al. (2022) [74]	Experimental in vitro + Genomic study using Next-gen sequencing	6 Chinese ESCC patients	(KYSE-150 and TE-1) ESCC, 2 cell lines	Oesophageal squamous cell carcinoma	X-ray	4Gy	X-ray irradiator	Key gene mutations associated with radiosensitivity	Identification of radioresponsive genes	To identify mutations in genes that correlate with radioresponse in ESCC	Whole-exome sequencing (WES), targeted gene analysis	Analysis of gene mutations and their correlation with radiosensitivity	Several key mutations in genes were identified that correlate with radiosensitivity in ESCC	Specific genetic mutations that could serve as biomarkers for predicting the response to radiation therapy	These findings could be useful for personalising radiotherapy in ESCC patients based on genetic profiling	Several radioresponsive genes, including TP53, EGFR, and ATM, NOTCH1	DNA damage response pathways, cell cycle regulation
Yang et al. (2005) [75]	Experimental in vitro comparative	4 human cancer cell lines	Human hepatoma cells (SMMC-7721), liver cells (L02), melanoma cells (A375) and cervical tumour (HeLa)	Various human cancer cell lines	Gamma radiation	0 Gy - 8 Gy	Co-60 source	Clonogenic survival, DNA damage, and apoptosis	Differential radiosensitivity among the cell lines. Frequency of chromatid breaks	Radiosensitivity comparison between tumour cells and normal liver cells	Clonogenic assay, γH2AX foci formation, apoptosis assay	Assessment of clonogenic survival and DNA damage post-irradiation	Tumour cells exhibited varying degrees of radiosensitivity, with normal liver cells showing higher resistance	Significant differences in radiosensitivity were observed among the tumour cell lines compared to normal liver cells	Importance of tumour-specific radiosensitivity in guiding radiotherapy strategies	N/A	N/A
Yoshida et al. (2009) [76]	Cohort genetic epidemiology study	124 Cases of Lung Ca and 2160 participants	Atomic bomb survivors	Human	Ionising radiation (bomb exposure)	<5 mGy	Environmental	Cancer development	EGFR polymorphism	None	Number of CA repeats in PCR from the peripheral blood sample	Unclear	Dose-dependent cancer risk	Genetic variation modifies risk	In vitro studies are required to establish the mechanistic link between the CA repeat polymorphism, EGFR production and radiation exposure in lung carcinogenesis	EGFR	N/A

Discussion

Definitional Ambiguity

This scoping review aimed to map and analyse how radiosensitivity and radiosusceptibility are defined, measured, and applied in the literature on ionising radiation. The analysis demonstrates that, despite substantial research into inter-individual radiation response, the two concepts are often inconsistently applied, with limited explicit distinction between them.

Many studies investigate radiosensitivity through cellular or molecular endpoints, but do not explicitly situate their findings within a broader conceptual framework. For example, Van Eerde et al. [73] compared three rat strains for the development of radiation-induced lung injury, revealing marked differences in tissue response that reflect intrinsic radiosensitivity at the organ level. Similarly, Popanda et al. [61] assessed DNA damage and repair in lymphocytes from breast cancer patients, showing correlations between cellular repair capacity and acute skin reactions to radiotherapy - again highlighting individual variation in immediate biological response without explicitly addressing long-term risk.

Other studies focus on outcomes more consistent with radiosusceptibility, particularly in the context of clinical or population-level endpoints. For instance, Golomb et al. [34] evaluated susceptibility to radiation-related adverse effects in veterans with Gulf War illness, demonstrating differential risk of clinical complications following similar exposures. Genetic association studies completed by Alsbeih et al. and De Ruyck et al. explored DNA repair gene variants in patients with gynaecological tumours, linking genetic variation to late clinical radiosensitivity, which reflects the continuum between immediate cellular response and long-term tissue- or organism-level susceptibility [20,29].

Several studies occupy an intermediate position, highlighting the biological overlap between radiosensitivity and radiosusceptibility. Mitsuhashi et al. [53] identified enhanced expression of radiation-induced CDKN1A mRNA in patients with multiple primary breast cancers, demonstrating a measurable cellular response that also correlates with potential long-term cancer risk. Similarly, Dulong et al. [31] observed that NFATC2 modulation in dermal fibroblasts corresponded with severe clinical side effects, illustrating how molecular radiosensitivity can manifest in clinically significant outcomes.

Despite these insights, the literature frequently lacks standardisation and conceptual clarity. Radiosensitivity is assessed using diverse endpoints - including DNA damage assays, apoptosis measurements, and gene expression profiles - while radiosusceptibility is often inferred from clinical outcomes without explicit definition [28,36,41,57]. This inconsistency complicates synthesis across studies and limits the ability to generalise findings [27], underscoring a persistent gap between mechanistic understanding and clinical application.

Collectively, the findings reinforce the premise that radiosensitivity and radiosusceptibility, while conceptually distinct, are closely intertwined in practice. Radiosensitivity primarily reflects immediate cellular and tissue responses, whereas radiosusceptibility captures the probability of adverse health outcomes, particularly stochastic effects such as cancer. The literature reveals both overlap and ambiguity, highlighting the need for more precise definitions and standardised approaches to measurement - an issue that forms the focus of subsequent sections in this discussion.

Conceptual Overlap

A central finding of this review is the complex and often indistinct boundary between radiosensitivity and radiosusceptibility. The studies analysed demonstrate that, in practice, they frequently overlap across biological scales. This overlap is particularly evident in studies linking early cellular damage to clinical outcomes. For example, Pinar et al. [60] reported that radiation-induced DNA damage in breast cancer patients was predictive of long-term toxicity following radiotherapy. Similarly, Pajic et al. [57] demonstrated significant inter-individual variability in lymphocyte response using cytogenetic assays, reinforcing the role of intrinsic cellular radiosensitivity. However, in both cases, these cellular endpoints are used to infer clinical consequences, thereby extending into the domain of radiosusceptibility.

Genetic association studies further illustrate this continuum. Reuther et al. [62] and Kelsey et al. [40] identified associations between specific single-nucleotide polymorphisms and both early and late radiation-induced effects, suggesting that genetic variation influences not only cellular response but also the likelihood of adverse clinical outcomes. Likewise, Smirnov et al. [68] demonstrated that inherited genetic variation contributes to differences in radiation-induced cell death, providing mechanistic insight into how radiosensitivity at the cellular level may underpin broader susceptibility.

The relationship between these concepts is also reflected in studies examining clinical endpoints such as toxicity and treatment response. For instance, Tsougos et al. [72] developed dose-response models predicting radiation-induced pneumonitis, linking radiation exposure parameters with observed clinical effects. While such models primarily address radiosusceptibility, they are inherently dependent on underlying biological responses characteristic of radiosensitivity. Similarly, Seo et al. [66] showed that accumulated genetic alterations influence response to radiotherapy, bridging tumour response, normal tissue effects, and broader susceptibility to radiation outcomes.

Population-based and epidemiological studies further complicate this distinction. Yoshida et al. [76] examined lung cancer susceptibility among atomic bomb survivors in relation to genetic polymorphisms and radiation dose, directly addressing radiosusceptibility. However, the underlying mechanisms - such as impaired DNA repair or increased genomic instability - are rooted in radiosensitivity. Likewise, Dong et al. [30] demonstrated that increased γ-radiation sensitivity was associated with elevated gastric cancer risk, reinforcing the link between cellular response and long-term disease development.

Additional studies highlight variability in radiation response across different biological systems and contexts. Brzozowska et al. [27] compared in vivo and in vitro radiosensitivity in both healthy individuals and cancer patients, revealing discrepancies between cellular assays and clinical outcomes. This finding underscores the challenge of using radiosensitivity as a direct surrogate for radiosusceptibility, as the relationship between the two is influenced by multiple modifying factors beyond intrinsic cellular response [4].

Collectively, these findings suggest that radiosensitivity and radiosusceptibility exist along a biological continuum rather than as discrete entities. Radiosensitivity represents the immediate mechanistic response to radiation exposure, including DNA damage and repair processes, while radiosusceptibility reflects the cumulative probability of adverse outcomes shaped by these initial responses in combination with genetic, environmental, and clinical factors [4].

Despite this interconnection, maintaining a conceptual distinction remains important. The use of cellular or molecular endpoints as proxies for long-term risk is common but not always justified, particularly given the variability observed between experimental systems and clinical outcomes. The lack of consistent terminology and clear definitions across studies further contributes to ambiguity, limiting the comparability of findings and the development of unified frameworks.

Molecular Determinants

A key theme emerging from this review is the central role of genetic and molecular factors in driving inter-individual variability in response to ionising radiation. Across the literature, variation in DNA repair capacity, gene expression, and inherited genetic polymorphisms consistently underpin differences in both radiosensitivity and radiosusceptibility [28,40,41,68]. These findings align with the growing field of radiogenomics, which seeks to link molecular characteristics with radiation response at both cellular and clinical levels.

At the cellular level, several studies highlight the importance of DNA damage response pathways in determining radiosensitivity. Bürger et al. [28] examined lymphocytes from individuals with Nijmegen breakage syndrome and demonstrated significantly increased radiation-induced DNA damage and impaired repair capacity compared to controls. This provides clear evidence that deficiencies in DNA repair mechanisms directly enhance radiosensitivity. Similarly, Howe et al. [38] investigated radiation-induced bystander effects and found that inter-individual variability in these responses correlated with intrinsic radiosensitivity, suggesting that not only direct DNA damage but also intercellular signalling pathways contribute to differential radiation responses.

Genetic variation has also been shown to influence radiation response across broader populations. Rosenberger et al. [63] demonstrated heritability of radiation response within lung cancer families, supporting the role of inherited genetic factors in determining individual radiosensitivity. In a complementary approach, Smirnov et al. identified specific genetic variants associated with radiation-induced cell death, further reinforcing the contribution of genetic background to cellular response variability [68]. These findings collectively indicate that radiosensitivity is not solely a function of environmental exposure but is significantly modulated by inherited traits.

At the clinical level, molecular determinants are increasingly linked to both treatment outcomes and adverse effects. Alsbeih et al. [21] identified polymorphisms in the HDM2 promoter as potential biomarkers associated with radiation toxicity, suggesting that genetic variation can predict susceptibility to adverse treatment effects. Similarly, Reuther et al. demonstrated associations between single-nucleotide polymorphisms in functional pathways and both early and late radiation-induced toxicity, bridging molecular mechanisms with clinical radiosusceptibility [62]. These studies highlight the potential for genetic profiling to inform risk stratification in radiotherapy.

Gene expression studies further support the role of molecular regulation in radiation response. Lee et al. [44] conducted proteomic analysis of head and neck cancer cell lines with differing radiosensitivity, identifying distinct protein expression patterns associated with radiation response. This suggests that differences in cellular phenotype, driven by underlying molecular pathways, contribute to variability in radiosensitivity. Likewise, Alchinova et al. [19] explored radioadaptive responses in human subjects and demonstrated variability in functional responses to radiation exposure, indicating that adaptive molecular mechanisms may also influence individual sensitivity.

Importantly, these molecular findings extend beyond immediate cellular effects to influence long-term outcomes. Dong et al. [30] reported that individuals with higher γ-radiation sensitivity exhibited an increased risk of gastric cancer, providing direct evidence linking molecular radiosensitivity to radiosusceptibility. Similarly, Yoshida et al. [76] demonstrated that genetic polymorphisms in the epidermal growth factor receptor gene modified lung cancer risk in radiation-exposed populations, reinforcing the connection between genetic variation and long-term disease risk.

Despite these advances, several challenges remain. The molecular determinants identified across studies are highly heterogeneous, involving multiple genes and pathways, including DNA repair, cell cycle regulation, apoptosis, and signal transduction [43,44,68,74]. This complexity makes it difficult to identify universal biomarkers of radiosensitivity or radiosusceptibility. Furthermore, many studies focus on single genes or pathways, which may not capture the multifactorial nature of radiation response.

Overall, the evidence indicates that genetic and molecular factors are fundamental to understanding individual variability in radiation response. These determinants operate across multiple biological levels, influencing both immediate cellular radiosensitivity and longer-term radiosusceptibility.

Measurement Challenges

A major challenge identified in this review is the lack of standardisation in the measurement of radiosensitivity and radiosusceptibility. Across the literature, a wide range of experimental, clinical, and modelling approaches are employed, often with limited comparability between studies [23,38,40,72]. This methodological heterogeneity complicates the interpretation of findings and represents a significant barrier to the integration of these concepts into clinical and radiation protection frameworks.

At the cellular level, radiosensitivity is most often assessed using cytogenetic and DNA damage assays. For example, Pajic et al. [57] compared the dicentric chromosome assay and micronucleus assay in human lymphocytes, demonstrating variability in radiation response between individuals depending on the method used. Similarly, Bürger et al. [28] utilised the Comet assay to quantify DNA damage and repair in patients with DNA repair deficiencies, highlighting increased sensitivity but also illustrating how results are assay-dependent. While these techniques provide valuable insight into cellular responses, differences in sensitivity, specificity, and endpoints limit their direct comparability.

Other studies employ functional or molecular endpoints, further contributing to variability in measurement. Alchinova et al. [19] assessed radioadaptive response as a functional indicator of radiation sensitivity, while Howe et al. [38] examined radiation-induced bystander effects, demonstrating that intercellular signalling responses may also serve as markers of radiosensitivity. However, these approaches measure distinct biological processes, making it difficult to establish a unified framework for assessing individual radiation response.

At the tissue and clinical level, radiosusceptibility is often inferred from observed outcomes such as toxicity or disease incidence. For instance, Tsougos et al. [72] developed dose-response models to predict radiation-induced pneumonitis based on clinical data, while Pinar et al. [60] linked DNA damage markers to long-term toxicity in breast cancer patients. Although these studies provide clinically relevant endpoints, they are influenced by numerous confounding factors, including treatment parameters, patient characteristics, and environmental exposures, making it challenging to isolate intrinsic susceptibility.

In addition, discrepancies between in vitro and in vivo assessments further complicate interpretation. Brzozowska et al. [27] demonstrated differences between cellular radiosensitivity measured in vitro and clinical outcomes observed in patients, suggesting that laboratory-based assays may not fully capture the complexity of whole-organism responses. This highlights the limitation of relying on single-method approaches to predict radiosusceptibility.

Genetic and genomic approaches introduce additional layers of complexity. Studies completed by Kelsey et al. [40] and Reuther et al. [62] use single-nucleotide polymorphisms and pathway analyses to assess radiation response, while others focus on gene expression or proteomic profiling. Although these methods offer promising insights, they often produce heterogeneous and sometimes inconsistent results, reflecting the multifactorial nature of radiation response and the influence of multiple interacting pathways.

**Figure 4:**
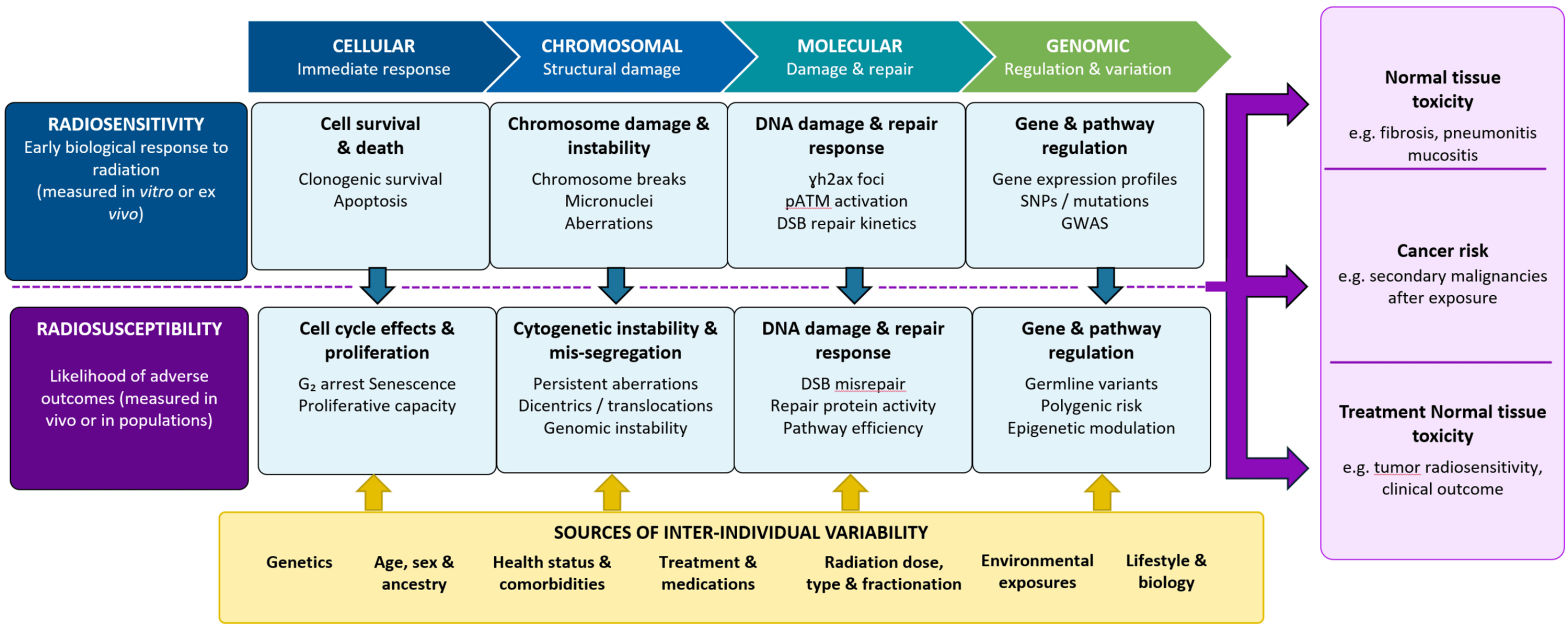
Overview of biological determinants of radiosensitivity and radiosusceptibility Schematic representation of key mechanisms underlying individual radiation response, highlighting processes from cell cycle dynamics and chromosomal damage to DNA repair, genetic variation, and population-level outcomes. Image Credit: Abdul Nadim Asil This image is an original, author-created schematic produced using CAD software, Blender 5.1 (Blender Foundation, Amsterdam, Netherlands), and Microsoft PowerPoint (Microsoft® Corp., Redmond, WA, USA), and was not generated using AI.

The findings of this review demonstrate that there is no universally accepted method for measuring radiosensitivity or radiosusceptibility. Instead, a diverse range of assays and models are used, each capturing different aspects of radiation response. This lack of standardisation limits the ability to compare results across studies, would hinder meta-analysis, and reduces the translational potential of research findings. These relationships and methodological disparities across biological levels are summarised in Figure 4.

Clinical Implications

The variability in radiosensitivity and radiosusceptibility identified across the literature has important implications for clinical practice, particularly in the context of radiotherapy. A consistent finding from the studies analysed is that individual differences in radiation response can significantly influence both treatment efficacy and the risk of adverse effects [24,59,60,66]. This highlights the potential value of incorporating biological and genetic information into clinical decision-making to support more personalised approaches to radiation treatment.

Several studies demonstrate that molecular and genetic factors can influence treatment outcomes. For example, Seo et al. [66] showed that accumulated alterations in driver and passenger genes are associated with differential responses to radiotherapy, suggesting that tumour and host genomic profiles may affect both radiosensitivity and treatment resistance. Similarly, Alsbeih et al. [21] identified polymorphisms in the HDM2 promoter as candidate biomarkers associated with radiation toxicity, indicating that genetic variation may help predict which patients are at greater risk of adverse effects. These findings support the emerging role of radiogenomics in guiding treatment planning.

Clinical modelling studies further emphasise the relevance of individual variability. Tsougos et al. [72] developed dose-response models for predicting radiation-induced pneumonitis, demonstrating how patient-specific and treatment-related factors can be integrated to estimate risk. While such models are primarily based on population-level data, their predictive capacity could be enhanced by incorporating individual radiosensitivity markers. Likewise, Kelsey et al. [40] identified associations between genetic polymorphisms and lung toxicity, reinforcing the importance of integrating biological variability into risk prediction models.

In addition to toxicity, variability in radiosensitivity may also influence tumour response. Ahmed et al. [18] reported differences in radiosensitivity among liver metastases depending on primary tumour histology, suggesting that intrinsic biological characteristics can affect treatment outcomes. Although this study focuses on tumour-level response, it highlights the broader principle that radiation response is not uniform and may vary across both tumour and normal tissues. Similarly, Lee et al. [44] demonstrated differential protein expression patterns in head and neck cancer cell lines with varying radiosensitivity, indicating that molecular characteristics can influence treatment response at the cellular level.

Despite these advances, the translation of radiosensitivity and radiosusceptibility into routine clinical practice remains limited. One reason for this is the lack of validated, standardised biomarkers that can reliably predict individual response. Although Pinar et al.’s and Pajic et al.’s studies demonstrate associations between cellular responses and clinical outcomes [57,60], these findings are not yet sufficiently robust or consistent to guide treatment decisions.

Translational Gap

Despite substantial advances in understanding radiosensitivity and radiosusceptibility at the molecular and cellular levels, a key finding of this review is the persistent gap between experimental research and clinical application. While numerous studies identify potential biomarkers and mechanisms underlying individual radiation response, relatively few have been successfully translated into routine clinical practice [23,24,36]. As illustrated in Figure 4, this gap reflects the challenge of linking cellular and molecular radiosensitivity with whole-organism radiosusceptibility and clinical outcomes.

One major barrier lies in the limited reproducibility and validation of proposed biomarkers. For example, early studies such as those performed by Barber et al. [24] and Ban et al. [23] demonstrated associations between chromosomal radiosensitivity in peripheral blood lymphocytes and normal tissue toxicity following radiotherapy. Similarly, Hamasaki et al. [36] utilised γH2AX assays to quantify DNA damage and identify inter-individual variability in radiosensitivity. While these approaches show promise, their predictive value remains inconsistent across populations and clinical settings, limiting their utility as reliable clinical tools.

Efforts to incorporate such assays into clinical decision-making have been explored but remain inconclusive. Mariano Ruiz De Almodovar et al. [49] proposed the use of DNA damage assays to individualise radiotherapy in breast cancer patients. However, the transition from experimental assay to clinical implementation is hindered by issues of standardisation, scalability, and integration into existing workflows.

At the molecular level, gene expression profiling has emerged as another promising approach, yet similar translational challenges persist. Kitahara et al. [41] identified gene expression signatures capable of classifying cervical cancers according to radiosensitivity, while Lemke et al. [45] demonstrated that stem cell marker profiling may predict radiotherapy response in glioblastoma.

In addition, experimental models often fail to fully capture the complexity of in vivo radiation response. For instance, Girard et al. [33] demonstrated heterogeneity in radiation response across chondrosarcoma cell lines, while Lanvin et al. [43] identified molecular pathways contributing to glioblastoma radioresistance. Although such in vitro studies are essential for mechanistic understanding, their relevance to patient outcomes is not always direct, contributing to the disconnect between laboratory findings and clinical application.

Emerging evidence also suggests that external factors, including treatment context and therapeutic interventions, may further complicate translation. Jost et al. [39] showed that kinase inhibitors can increase radiation sensitivity in normal cells of cancer patients, indicating that radiosensitivity is not a fixed intrinsic property but can be modulated by concurrent treatments. This adds an additional layer of complexity to the use of radiosensitivity as a predictive biomarker in clinical settings.

Animal and genetic studies reinforce the importance of biological context but also highlight translational limitations. Haston et al. [37] identified both universal and radiation-specific genetic loci influencing susceptibility to pulmonary fibrosis in mice, while Lu et al. [48] demonstrated that ATM haploinsufficiency increases susceptibility to carcinogen-induced tumours. Although these findings provide strong evidence for genetic contributions to radiosusceptibility, differences between model systems and human populations limit direct clinical extrapolation.

Finally, variability between assay types and endpoints further impedes translation. Mayo et al. [50] assessed radiosensitivity in lung cancer patients using fluorescence in situ hybridisation, while other studies employ cytogenetic, molecular, or clinical endpoints. The lack of a standardised “gold standard” for measuring radiosensitivity or radiosusceptibility makes it difficult to compare findings and validate predictive models across studies.

Population Variability

A key finding across the literature is the substantial variability in radiation response observed both within and between populations. This inter-individual variability reflects the combined influence of genetic, biological, and environmental factors and underpins both radiosensitivity and radiosusceptibility. Figure 4 highlights how these determinants operate across multiple biological levels to produce heterogeneous radiation responses.

At the cellular level, variability is evident even among apparently similar individuals. Müller et al. [55] demonstrated significant differences in lymphocyte radiosensitivity between healthy individuals and cancer patients using the comet assay, indicating that intrinsic biological differences exist irrespective of disease status. Similarly, Shahidi et al. [67] reported variation in DNA repair kinetics between prostate cancer patients and healthy controls, further supporting the role of individual biological factors in modulating radiosensitivity. These findings are reinforced by Pajic et al. [57], who observed inter-individual variability in cytogenetic damage following radiation exposure, even under controlled experimental conditions.

Genetic predisposition represents a major contributor to this variability. Schmitz et al. [65] demonstrated heritability in radiation-induced apoptosis of lymphocyte subpopulations, providing strong evidence that radiosensitivity has a genetic basis. This is consistent with findings from Rosenberger et al. [63], which showed familial aggregation of radiation response, and Peng et al. [58], who highlighted the role of genetic susceptibility in radiation effects relevant to space exposure. Together, these studies suggest that inherited factors play a central role in determining both radiosensitivity and longer-term radiosusceptibility.

Variability is also observed across disease states and clinical populations. Milenkova et al. [52] reported increased chromosomal radiosensitivity in patients with multiple sclerosis, suggesting that underlying pathological conditions may influence radiation response. Similarly, Ban et al. [23] demonstrated differences in lymphocyte radiosensitivity among patients with different cancer types, including breast, head and neck, and cervical cancers. These findings indicate that both disease-specific and patient-specific factors contribute to heterogeneity in radiation response.

At the tumour level, heterogeneity in radiosensitivity is also well documented. Nakajima et al. [56] used patient-derived spheroids of cervical carcinoma to demonstrate variability in radiation response between tumours, while Steffen et al. [69] observed differences in radiosensitivity among HER2-overexpressing cell lines. Likewise, Girard et al. [33] highlighted heterogeneity in response to different radiation modalities (X-rays versus carbon ions) across chondrosarcoma cell lines.

Molecular and proteomic studies further illustrate the complexity of inter-individual variation. Pietrowska et al. [59] identified associations between plasma proteome profiles, DNA repair capacity, and acute mucosal reactions in head and neck cancer patients, suggesting that systemic biological differences contribute to clinical radiosusceptibility. Similarly, Xu et al. [74] identified key mutations in radioresponsive genes in oesophageal cancer, reinforcing the role of tumour-specific genetic alterations in modulating radiation response.

Importantly, variability is not limited to intrinsic biological factors but is also influenced by external and treatment-related variables. Differences in radiation dose, fractionation, concurrent therapies, and environmental exposures all contribute to observed heterogeneity. This is consistent with findings from Tsougos et al. [72], where clinical outcomes such as pneumonitis were influenced by both dosimetric and patient-specific factors, highlighting the multifactorial nature of radiosusceptibility.

Review Limitations

While the studies included in this review provide valuable insight into radiosensitivity and radiosusceptibility, several important limitations must be considered when interpreting the findings. These limitations relate to study design, methodological heterogeneity, sample size, and the broader challenges of translating experimental findings into clinically meaningful conclusions.

A primary limitation across the literature is the substantial heterogeneity in study methodologies and endpoints. As highlighted in earlier sections, radiosensitivity is assessed using a wide range of assays, including micronucleus formation [23], comet assays [55,67], γH2AX foci [36], and gene or protein expression profiling [41,44]. While each method captures specific aspects of radiation response, the lack of standardisation makes direct comparison between studies difficult and limits the ability to synthesise findings quantitatively. This variability also contributes to inconsistent conclusions regarding the predictive value of different biomarkers.

Another key limitation is the frequent reliance on small sample sizes and highly selected populations. Many studies, particularly those involving molecular or cellular analyses, are conducted on limited cohorts or specific patient groups. For example, Lemke et al. [45] and Nakajima et al. [56] utilise tumour-derived models with relatively small sample numbers, which may not be representative of broader patient populations. Similarly, studies focusing on rare conditions or specific subgroups, such as those completed by Milenkova et al. [52], may limit generalisability. This raises concerns regarding statistical power and the robustness of reported associations.

An additional limitation relates to the methodological approach of the present scoping review. Consistent with the objectives of scoping review methodology, the included studies were mapped and synthesised descriptively, and no formal assessment of methodological quality or risk of bias was undertaken. Consequently, studies of varying methodological rigour were considered equally during evidence mapping, and the findings presented should not be interpreted as an assessment of the strength or quality of the underlying evidence. Furthermore, the synthesis was primarily qualitative in nature due to substantial heterogeneity in study designs, populations, endpoints, and outcome measures. This precluded quantitative comparison between studies and may have introduced an element of interpretive subjectivity when identifying overarching themes and conceptual relationships. While these approaches are appropriate for a scoping review, they limit the ability to draw definitive conclusions regarding causality, effect size, or the comparative validity of specific biomarkers and assessment methods.

The use of in vitro and ex vivo models represents another important limitation. While such models are essential for mechanistic investigation, they do not fully replicate the complexity of in vivo biological systems. Girard et al. [33] and Lanvin et al. [43] demonstrated variability in radiation response in cell lines, yet these findings may not directly translate to clinical outcomes due to the absence of systemic factors such as immune response, microenvironment, and whole-organism interactions. This limitation contributes to the broader translation gap identified in the above sections.

In addition, many studies infer radiosusceptibility from surrogate endpoints rather than direct clinical outcomes. For instance, cytogenetic damage or DNA repair capacity is often used as a proxy for long-term risk, as seen in Pajic et al. [57] and Pinar et al. [60]. While these measures provide important insight into radiosensitivity, their relationship with long-term outcomes such as cancer development is not always clearly established. This may lead to overinterpretation of cellular findings in the context of population-level risk.

Confounding factors also represent a significant challenge. Clinical outcomes following radiation exposure are influenced by multiple variables, including age, sex, comorbidities, treatment parameters, and environmental exposures. For example, Tsougos et al. [72] demonstrated that radiation-induced pneumonitis is influenced by both dosimetric and patient-related factors. Similarly, Jost et al. [39] showed that concurrent treatments such as kinase inhibitors can modify radiosensitivity. Failure to adequately control for these variables may bias study findings and limit the ability to isolate intrinsic radiosensitivity or radiosusceptibility.

Another limitation is the inconsistency in terminology and conceptual definitions across studies. As identified in earlier sections, the terms radiosensitivity, radiosusceptibility, and radiation response are often used interchangeably without clear distinction. This lack of conceptual clarity complicates interpretation and synthesis of the literature and may contribute to variability in reported findings.

Finally, there is a lack of large-scale, longitudinal studies linking molecular or cellular markers to long-term clinical outcomes. While studies such as Dong et al. and Yoshida et al. provide valuable epidemiological evidence [30,76], most research remains focused on short-term or intermediate endpoints. This limits the ability to fully understand the relationship between radiosensitivity and radiosusceptibility over time.

Overall, these limitations highlight the need for more standardised methodologies, larger and more representative study populations, and integrated approaches that link mechanistic findings with clinical outcomes.

Implications for Definitions

A central objective of this scoping review was to examine how the terms radiosensitivity and radiosusceptibility are defined and applied within the literature. The findings highlight a persistent lack of conceptual clarity, with many studies using these terms inconsistently or interchangeably despite their distinct theoretical meanings.

Across the studies analysed, radiosensitivity is most operationalised through measurable biological responses at the cellular or tissue level. These include DNA damage, chromosomal aberrations, apoptosis, and gene expression changes, as demonstrated in the studies discussed above [36,41,55,57]. In contrast, radiosusceptibility is typically inferred from clinical or epidemiological outcomes, including normal tissue toxicity, cancer risk, and long-term adverse effects [30,72,76]. However, many studies do not explicitly distinguish between these endpoints, instead using cellular markers as proxies for long-term risk without clearly defining the conceptual transition between the two.

This lack of distinction is further reinforced by studies that inherently span both domains. For example, Barber et al. [24] and Pinar et al. [60] link cellular measures of DNA damage or chromosomal radiosensitivity with clinical outcomes such as late toxicity, effectively bridging radiosensitivity and radiosusceptibility.

The findings of this review suggest that radiosensitivity and radiosusceptibility should be understood as related but distinct constructs within a continuum of radiation effects. Importantly, the relationship between these concepts is not purely hierarchical but interactive. While radiosensitivity may contribute to radiosusceptibility, it is not the sole determinant. As demonstrated by Brzozowska et al. [27] and Müller et al. [55], variability in cellular response does not always directly correspond to clinical outcomes. This indicates that radiosusceptibility is influenced by additional factors, including genetic background, environmental exposures, and clinical context, which extend beyond intrinsic cellular radiosensitivity.

Without clear conceptual boundaries, it becomes difficult to compare findings across studies, synthesise evidence, or develop standardised assessment tools. This issue is particularly evident in the use of biomarkers, where cellular indicators of radiosensitivity are often proposed as predictors of radiosusceptibility without sufficient validation [23,57].

Based on the findings of this review, there is a clear need for more precise and standardised definitions of radiosensitivity and radiosusceptibility. One potential approach is to explicitly define these terms according to the level of biological organisation and type of outcome being measured. For example: Radiosensitivity: the extent of immediate biological response to ionising radiation at the cellular or tissue level, including DNA damage, repair capacity, and cell survival. Radiosusceptibility: the likelihood of developing adverse health outcomes following radiation exposure, particularly stochastic effects such as cancer, at the individual or population level.

Adopting such definitions would help to facilitate more consistent application of these terms across studies. It would also support the development of integrated models that link mechanistic understanding with clinical outcomes, as conceptually represented in Figure 4.

Future Directions

The findings of this review highlight several important avenues for future research aimed at improving the understanding and application of radiosensitivity and radiosusceptibility. While substantial progress has been made in identifying biological determinants of radiation response, significant gaps remain in translating these insights into clinically meaningful and standardised approaches.

A key priority is the development of integrated, multi-level models of radiation response that combine molecular, cellular, and clinical data. Many existing studies focus on single endpoints - such as DNA damage [36], cytogenetic alterations [57], or gene expression profiles [41] - but do not account for the complex interactions between these levels. Future research should aim to integrate genomic, proteomic, and functional data, as demonstrated in part by Pietrowska et al. [59], to develop more comprehensive models capable of predicting both radiosensitivity and radiosusceptibility. Figure 4 provides a conceptual framework for such multi-level integration.

Closely related to this is the need for large-scale, well-designed prospective studies. Much of the current evidence is derived from small cohorts or experimental systems, limiting generalisability. Studies by Yoshida et al. [76] and Dong et al. [30] demonstrate the value of population-based approaches in linking radiation exposure to long-term outcomes, but similar large-scale efforts incorporating molecular biomarkers are still lacking. Future research should prioritise longitudinal designs that follow individuals from exposure through to clinical outcomes, enabling more robust validation of predictive markers.

Standardisation of methodologies also represents a critical area for development. As highlighted throughout this review, a wide range of assays are used to assess radiosensitivity, including comet assays [55], micronucleus assays [23], and γH2AX-based techniques [36]. The lack of a universally accepted “gold standard” limits comparability between studies.

Another important direction is the refinement and validation of predictive biomarkers for clinical use. Genetic and molecular markers identified in studies completed by Alsbeih et al. [21], Reuther et al. [62], and Smirnov et al. [68] show promise for predicting radiation response but require further validation in diverse populations. In addition, emerging research on gene expression [66], proteomics [59], and mutational profiling [74] suggests that multi-marker approaches may be more effective than single biomarkers in capturing the complexity of radiation response.

The role of tumour heterogeneity and the tumour microenvironment also warrants further investigation. Studies by Girard et al. [33], Ahmed et al. [18], and Nakajima et al. [56] demonstrate significant variability in tumour radiosensitivity, which has direct implications for treatment outcomes. Future research should explore how tumour-specific factors interact with host radiosensitivity to influence overall response to radiotherapy, particularly in the context of emerging treatment modalities.

In addition, there is a need to better understand modifiable factors influencing radiosensitivity. Jost et al. [39] showed that kinase inhibitors can alter radiation sensitivity in normal cells, highlighting the potential for therapeutic modulation of radiation response. Further research into such interactions could inform strategies to enhance tumour radiosensitivity while protecting normal tissues, thereby improving the therapeutic ratio.

Another promising area is the application of advanced computational approaches, including machine learning and predictive modelling. Tsougos et al. [72] demonstrated the utility of dose-response models, but future work could incorporate high-dimensional biological data to develop more accurate and individualised predictive tools. These approaches may play a key role in translating complex biological data into clinically actionable insights.

Finally, future research should prioritise clearer conceptual frameworks and consistent terminology. As identified in this review, ambiguity in the use of radiosensitivity and radiosusceptibility limits the comparability and interpretability of studies. Establishing consensus definitions and aligning research methodologies with these definitions will be essential for advancing the field.

In summary, future research should focus on integrating multi-level data, standardising methodologies, validating predictive biomarkers, and strengthening links between mechanistic studies and clinical outcomes. Addressing these priorities will be critical for improving the understanding of individual radiation response.

## Conclusions

This scoping review examined how radiosensitivity and radiosusceptibility are defined and applied within studies of ionising radiation. The findings demonstrate that, although both concepts are widely used, they are often inconsistently defined and frequently overlap in practice. Radiosensitivity is most appropriately understood as the immediate biological response to radiation at the cellular or tissue level, while radiosusceptibility reflects the longer-term risk of adverse outcomes, particularly stochastic effects such as cancer. However, many studies use cellular markers as proxies for clinical risk without a clear distinction, contributing to conceptual ambiguity.

Across the literature, substantial inter-individual variability in radiation response is evident, driven by genetic, molecular, and environmental factors. Despite advances in identifying potential biomarkers and mechanisms, translation into clinical practice remains limited due to methodological heterogeneity, lack of standardisation, and insufficient validation. Greater clarity in definitions, alongside integrated and standardised approaches, will be essential for improving understanding and enabling the development of personalised radiotherapy. Beyond clinical applications, a more robust distinction between radiosensitivity and radiosusceptibility may also enhance radiation protection frameworks and individualised radiation risk assessment by improving the identification of populations at increased risk of radiation-induced adverse effects, including cancer. Continued efforts to harmonise terminology, methodologies, and outcome measures will be critical for advancing both radiobiological research and evidence-based radiation protection practices.
